# EQ Health and Wellbeing EQ-HWB: A Psychometric Assessment Across 6 Conditions and the General Population in the United Kingdom

**DOI:** 10.1016/j.jval.2025.07.028

**Published:** 2025-12

**Authors:** Anju Devianee Keetharuth, Clara Mukuria, Tessa Peasgood, Allan Wailoo

**Affiliations:** 1 Sheffield Centre for Health and Related Research, University of Sheffield, Sheffield, England, UK

**Keywords:** EQ-HWB, general population, patient sample, psychometric performance, validation

## Abstract

**Objectives:**

The EQ Health and Wellbeing (EQ-HWB) tools have been developed to measure and value outcomes of both health and social care interventions, including those of carers, in a manner suitable for use in economic evaluation. The aim of this article is to add to the body of psychometric evidence for the performance of EQ-HWB, and its shorter version EQ-HWB-9, by assessing construct validity and reliability.

**Methods:**

A sample of patients (*n =* 767) across 6 broadly defined health conditions and a sample of the general population (*n =* 302) completed the EQ-HWB measures alongside other measures. Convergent validity was assessed using Spearman and Pearson correlations. Known-group validity was investigated by using several self-reported variables and disease specific questions for the patient sample. Test-retest reliability was assessed by intraclass correlation coefficients and the kappa statistic.

**Results:**

Convergent validity between EQ-HWB items and related items from EQ-5D-5L, SWEMWBS, and ICECAP-A was highest in the patient sample. At the scale level, the highest correlations of EQ-HWB summative score and other measures were observed with both PHQ-8 and GAD-7 followed by EQ-5D-5L and ICECAP-A. The EQ-HWB measures showed ability to detect differences in the defined known groups. Comparing across measures, the EQ-HWB measures had the highest standardized effect sizes for groups defined by emotional problems. The EQ-HWB measures were found to be reliable with test-retest reliability being >0.8 for both groups.

**Conclusions:**

The results show that the EQ-HWB measures have promising psychometric properties across both the patient and general populations.

## Highlights

• This study adds to evidence on the psychometric performance of EQ-HWB and the shorter EQ-HWB-9 capturing health, social care, and carer-related quality of life by assessing construct validity and reliability in UK health condition and general population samples.
• The measures showed strong test-retest reliability at the scale level and effectively distinguished between all tested known groups with large effect sizes—something none of the 5 comparison measures (EQ-5D-5L, SWEMWBS, ICECAP-A, PHQ-9, and GAD-7) achieved.
• The broad domains in the EQ-HWB, designed for social care users and carers in addition to patients, do not compromise its ability to differentiate based on physical and mental health.

## Introduction

Many international health technology assessment organizations such as the National Institute of Health and Care Excellence in England use economic evaluation, the comparative assessment of the costs and benefits of alternative interventions, to support resource allocation decisions in healthcare.^1^ Benefits can be assessed using quality-adjusted life-years, which combine length of life with health-related quality of life, captured using utility values, into a single metric. Utility values for health-related quality of life are typically generated using generic, preference-weighted measures, which can be applied to a wide range of disease areas.^2^ There are different generic preference-weighted measures that can be used in economic evaluation. For consistency, National Institute of Health and Care Excellence recommend EQ-5D, which is one of the most widely used generic preference-weighted measures.^1^ However, health measures may be limited in their ability to assess the benefits of health technologies or policies in some patient populations.^3^ They may not cover aspects of benefit that are important in contexts such as social care, such as autonomy and independence.^4^ Conditions and interventions may also have an impact on informal carers, such as family members providing informal care, including aspects beyond their health.

Other measures have been developed to capture wider benefits beyond health or for use in specific populations in which aspects beyond health are required. For example, the Adult Social Care Outcomes Toolkit (ASCOT) was developed to measure social-care-related quality of life focusing on outcomes that were identified as important for social care users.^4^ Informal care measures have also been developed to assess care-related outcomes such as the ASCOT-Carer^5^ and the Carer Experience Scale.^6^^,^^7^ In situations in which outcomes of interventions, for example a gardening club for the elderly, include social care-specific outcomes, such as increased independence in addition to improved health outcomes, could lead to double counting, and each alone excludes potentially important benefits. In recognition of this issue, Mulhern et al attempted to combine EQ-5D-5L and ASCOT in an online discrete choice experiment,^8^ but this approach remains exploratory. To address this, the EQ Health and Wellbeing (EQ-HWB), a measure of health, social care and carer-related quality of life has been developed to support decision-making across health and social care, including for informal carers.^9^ There are 2 main versions of the EQ-HWB measures, the 25-item measure (EQ-HWB) and a shorter version (EQ-HWB-9), consisting of 9 items.

The EQ-HWB measures currently hold “experimental version” status meaning they are available for researchers for validation and are potentially subject to minor future modifications.^10^ Recent validity testing suggests that the experimental version of the EQ-HWB-9 performs at least as good as the EQ-5D-5L in terms of capturing the impact of health conditions in the general population in the United Kingdom,^11^ in Italy,^12^ and in China.^13^ Similarly, the EQ-HWB has been found to perform well in general population samples in Australia^14^ and Ireland.^15^

Additionally, the EQ-HWB-9 has been shown to capture carers quality of life in Australia,^16^, ^17^, ^18^ in the United States,^19^ and in China.^20^

Test-retest based on small sample (*n* = 25) of carers in Australia^18^ and a small sample (*n* = 32) of patients with breast cancer in Indonesia^21^ found promising results at the instrument level. EQ-HWB and EQ-HWB-9 also showed good construct validity and responsiveness for this group of patients with cancer and compared favorably with FACT-8D, SWEMWBS, and EQ-5D-5L. Gaps remain in terms of evidence on reliability, particularly at the item level for the full EQ-HWB, and in terms of known group validity across a broad range of patient groups and social care needs.

This study aimed to assess the EQ-HWB measures in terms of their psychometric properties. Study objectives were to assess the construct validity and reliability of the EQ-HWB and EQ-HWB-9 and compare the EQ-HWB measures with other measures of health and social care quality of life.

## Methods

### Sample and Recruitment

A mixed population sample was targeted comprising of individuals with 6 self-reported health conditions (*n* = 767) and members of the general population who do not report having any of the 6 conditions (*n* = 302). Six condition groups were specifically targeted (*n* = 150 in each group) to represent mental health (anxiety and depression) and physical health (diabetes, arthritis, asthma, and chronic obstructive pulmonary disease [COPD]). These were chosen because they are conditions with a high prevalence in the United Kingdom and collectively cover a broad range of expected symptoms and quality-of-life impacts. For the general population sample, a broadly representative sample was recruited in terms of age (18-40 years 30%, 41-60 years 30%, and over 60 years 40%), sex (male: female 50:50), and ethnicity (80% white and 20% other) using proportions from the 2021 Census. Participants from the general population were asked whether they had received a diagnosis from a healthcare professional of a long-standing illness (troubled or likely to trouble them for at least 12 months). Both groups were asked to select all long-standing conditions that they had been diagnosed with, and if more than 1 condition, participants were asked to select the one that has the biggest impact on them.

Recruitment was undertaken online using an existing online panel that recruits individuals who are interested in taking part in research who then receive points for taking part that can be redeemed into shopping vouchers. Panel members were invited to take part in the study by completing a screener survey to identify eligibility. Eligible panel members saw the information sheet and then completed consent before completing the questionnaire. A randomly selected subsample of those who completed the questionnaire (from both the health condition and the general population samples) were invited to complete selected measures from the questionnaire after 1 week (*n* = 333) (for Survey, see Appendix 1 in Supplemental Materials found at https://doi.org/10.1016/j.jval.2025.07.028).

### Measures

#### EQ-HWB

The EQ-HWB^22^^,^^23^ consists of 25 questions which represent 23 subdomains that can be grouped into 7 domains: (1) activity/functioning (vision, hearing, mobility, daily activities, meaningful activities, self-care); (2) cognition (memory, concentrating/thinking clearly); (3) feelings and emotions (anxiety, safety, frustration, sad/depressed, hopelessness [nothing to look forward to]); (4) relationships (loneliness, support, stigma); (5) Autonomy, coping control (coping, control); (6) self-worth (felt good about self); and (7) physical sensations (fatigue, sleep, physical pain, discomfort).

The EQ-HWB-9 uses 9 questions across 6 domains excluding self-worth (mobility, daily activity, exhaustion, loneliness, concentration/thinking clearly, anxiety, sad/depressed, control, and pain). In this study, participants completed the EQ-HWB, and the EQ-HWB-9 was extracted from the longer measure.

The recall period is 7 days. Questions have different response options including difficulty (no, slight, some, a lot, and unable), frequency response options (none of the time, only occasionally, sometimes, often, and most or all of the time), and severity (no, mild, moderate, severe, and very severe). Pain and discomfort had questions with both frequency and severity response options. All items were scored from 1 (no difficulty) to 5 (highest level of difficulty). Both EQ-HWB measures were scored by summing across the response options with higher scores indicating poor health and well-being. The possible scores for EQ-HWB and EQ-HWB-9 range between 25 and 125 and 9 and 45 respectively. Additionally, the EQ-HWB-9 was scored as utilities (referred to as EQ-HWB-S utilities) based on the feasibility valuation study in the United Kingdom.^24^ These utilities range from -0.384 to 1.

### Other Measures

Other validated measures were included to provide comparisons with the EQ-HWB and to assess severity in the populations included in the study. All participants were asked to complete several core measures, including the health-related quality-of-life measure EQ-5D-5L,^25^^,^^26^ the mental well-being measure SWEMWBS Short Warwick Edinburgh Mental Wellbeing Scale,^27^ which aims to reflect functioning well and feeling good, the capability measure ICECAP ICEpop CAPability measure for Adults,^28^^,^^29^ the University of California, Los Angeles (UCLA) 3-item loneliness scale,^30^ the social-care related quality-of-life measure ASCOT,^4^^,^^31^ the Patient Health Questionnaire 8 (PHQ-8),^32^ and the Generalized Anxiety Disorder 7 (GAD-7).^4^^,^^30^^,^^31^^,^^33^ Details on the measures can be found in Table 1.^4^, ^24^, ^25^, ^29^, ^34^

**Table 1:** Measures used.

Measure	No of items	Domains/themes	Response options type (number)	Recall period	Scores used	Min and max score	Cutoffs
EQ-HWB	25	7 (activity/functioning; cognition; feelings and emotions; relationships; autonomy/coping/control; self-worth; physical sensations)	Frequency, difficulty and severity (5)	7 days	Summed scores have been used for analysis purposes (but are not recommended for general scoring)	25 and 125 (higher scores poorer health)	NA
EQ-HWB-9	9	6 (activity/functioning; cognition; feelings and emotions; relationships; autonomy/coping/control; physical sensations)	Frequency, difficulty and severity (5)	7 days		9 and 45 (higher scores poorer health)	NA
EQ-5D-5L	5	5 (mobility, self-care, usual activities, pain/discomfort, anxiety/depression)	Severity (5)	Today	Mapped from ΕQ-5D-5L to EQ-5D-3L†	−0.577 and 0.987	NA
SWEMWBS	7	1 (positive affect of mental well-being)	Frequency (5)	2 weeks	Both summative and Rasch scores	7 to 35 Rasch conversion scores as per user manual	NA
ICECAP-A	5	5 (capability; stability; attachment; achievement; autonomy; enjoyment)	Likert (4)	Moment	Preference weights‡	0 (no capability) 1 (full capability)	NA
PHQ-8	8	1 (depression), this excludes the question on suicide	Frequency (4)	2 weeks	Summed scores as per user manual	0 to 24 (higher scores more impaired health)	(0-4) minimal, (5-9) mild (10-14), moderate (15-24) severe §
GAD-7	7	1 (generalized anxiety)	Frequency (4)	2 weeks	Summed scores as per user manual	0 to 21 (higher scores more impaired health)	(0-4) minimal, (5-9)mild (10-14), moderate, or severe (15-21)
ASCOT	9	8 (control over daily life; personal cleanliness and comfort; food and drink; personal safety; social participation and involvement; occupation; accommodation cleanliness and comfort; dignity)	Which statement/situation best describes (4)	NA	Preference weights∥	−0.17 to 1	NA
UCLA	3	1 (loneliness), lack companionship, feel left out, feel isolated	Frequency (3)	NA	Summed scores as per user manual	3-9 (higher scores higher loneliness)	Not lonely (score <6) Lonely (score >6)¶

NA indicates not applicable.

† Reference: Hernández Alava et al.^25^

‡ Reference: Flynn et al.^29^

§ Given the low n in the original categories, the 2 mild categories were combined as 1 category and the (15-19) moderately severe and^20^, ^21^, ^22^, ^23^, ^24^ severe categories were also combined into 1 category in the analyses.

∥ Reference: Netten et al.^4^

¶ Reference: Surkalim et al.^34^

Participants completed questions about themselves, including about their health, education, and employment status before completing the other measures. The full survey is presented in Appendix 1 in Supplemental Materials found at https://doi.org/10.1016/j.jval.2025.07.028. The questionnaires completed by all participants in both samples were always presented first to the participants. EQ-HWB, SWEMWBS, ICECAP-A, and EQ-5D-5L were presented in a random order to participants. After 1 week, the subset of participants received a shorter questionnaire, which included the EQ-HWB, the EQ-5D-5L, and the SWEWMBS, as well as questions regarding symptoms and changes over the week. Appendix Table 1 in Supplemental Materials found at https://doi.org/10.1016/j.jval.2025.07.028 summarizes the various measures collected at different time points.

#### Distribution of scores

We used details on the distribution of scores (frequency endorsement and histograms) to identify whether there are ceiling effects for the general population and the health condition sample.

#### Construct validity

We examined 2 forms of construct validity: convergent and known-group validity. For convergent validity, convergence between EQ-HWB measures and 4 other measures (EQ-5D-5L, SWEMWBS, ICECAP-A, and ASCOT), was assessed. At score level, it is expected that although the measures measure different constructs, EQ-HWB would be moderately to highly correlated with these measures. First convergent validity using scores of the various measures was assessed by Pearson’s product moment correlation coefficients. Scores were also correlated with the arthritis pain visual analog scale (how severe is your arthritis pain today?) and a calculated index of severity for those with rheumatoid arthritis. Second, at item level, we identified the items from the EQ-5D-5L, SWEMWBS, ICECAP-A, and ASCOT that are most likely to correlate with the EQ-HWB items based on the wording of the items. We then hypothesized the strength of correlation for both samples before computing Spearman correlation coefficients. A correlation coefficient of ≥ 0.7 is taken as strong evidence of construct validity with the additional categories: ≤0.4, weak correlation and >0.4 to <0.7 as moderate correlation.^35^

Known-group validity was examined in terms of whether the EQ-HWB, the EQ-HWB-9 level sum score, and EQ-HWB-S utility score were able to discriminate between groups hypothesized to have different levels of health and well-being. For both samples of patients and the general population, we investigated known-group validity by using several self-reported variables, including overall assessment of general health, physical health, emotional health, UCLA loneliness scale scores, PHQ-8, GAD-7, presence of a long-term condition, and a count of conditions. Known-group validity was also assessed in populations across a variety of specific conditions: anxiety/depression, diabetes, arthritis, rheumatoid arthritis, and other respiratory conditions (asthma, COPD, and bronchitis), comparing groups based on severity of condition symptoms. Details of the various known groups including definitions and corresponding hypothesis and hypothesised strength of correlation are presented in Appendix Tables 2A-2E in Supplemental Materials found at https://doi.org/10.1016/j.jval.2025.07.028.

Differences were quantified using standardized effect sizes (SES) across severity subgroups calculated as the difference in mean scores between groups divided by the standard deviation of the milder of the 2 subgroups. In the case of 2 groups, Glass’s delta is used in favor of other measures of effect sizes because this does not require the assumption of equal variance between the groups; however, this is very similar to Cohens D and standard rules of thumb for judging effect sizes can still be applied: 0.2 to 0.49 considered small, 0.5 to 0.79 moderate, and ≥0.8 large.^20^ Construct validity was only assessed using baseline data. In cases which there were more than 2 known groups, Kruskal Wallis tests were used given the nonnormal distributions of the main measures.^36^

#### Reliability

Test-retest reliability was assessed on a subsample of 300 participants (from 330 invited) who completed the EQ-HWB measure between 1 and 2 weeks apart. From this subsample, 157 patients and 38 members of the general population reported an unchanged health-related quality of life at both administrations when asked the following question: “Thinking about your life in general, have you experienced any negative or positive changes over the past week that have had an impact on how you feel?” The response options for the latter question were no change, small changes, moderate changes, and big changes. Reliability was assessed by the intraclass correlation coefficient (ICC), in which an ICC > 0.8 would indicate very good test-retest reliability.^37^ At the item level, test-retest reliability was assessed using the kappa statistic in which the level of agreement is assessed as follows: 0 to 0.2: slight; 0.21 to 0.4: fair; 0.41 to 0.60:moderate; 0.61 to 0.80: substantial; and 0.81 to 1: almost perfect.^38^

## Results

### Sample Description

The demographics are reported in Table 2 below. A total of 1069 individuals in the United Kingdom were recruited, of whom 767 and 302 were from a health condition and general population sample, respectively. The health condition sample were recruited across 6 disease categories: anxiety, depression, diabetes, arthritis, asthma, and COPD. In some of the analyses that follow, asthma and COPD are grouped as respiratory conditions. Mean age in years (SD) of participants was 57.3 (14.1) for the health condition sample and 53.3 (14.0) for the general population. The percentage of female participants were 53.2% and 52.3% for the health condition sample and the general population sample respectively. The health condition sample was predominantly white (92%), but in the general population sample, people from ethnic minority backgrounds were more highly represented. Over 60% were married, and over 43% had a degree in each group. Around 66% of the general population was employed, and it was only 40% in the patient population. In the last column of the table, we provide some comparative data to add some context to the sample recruited in this study. Data on age, sex, ethnicity, marital status, education, and employment status for the general population of the United Kingdom were obtained from the 2021 Census conducted in England and Wales.^39^ Prevalence rates for the health conditions within the general population for England and Wales were obtained from the Quality Outcomes Framework.^40^

**Table 2:** Sample characteristics.

Characteristics	Description categories	Health condition (*n =* 767) %	General population (*n =* 302) %	QOF prevalence rates∗
Condition	Anxiety	19.82		NA
	Depression	19.56		12.29
	Diabetes	20.08		7.11
	Arthritis	20.47		0.77
	Asthma	12.91		6.38
	COPD	7.17		1.93
	Presence of a long-standing physical/mental health, illness/disability diagnosis	100	15.89	NA
				England and Wales general population†
Age group	18-40	16.95	25.17	37.69
	41-60	37.16	40.4	33.12
	61+	45.89	34.44	29.18
Sex	Female	53.19	52.32	51.04
	Male	46.81	47.68	48.96
Ethnicity	White British	90.22	68.21	81.7
	White non-British	1.43	2.98	
	Asian / Asian British	4.3	13.25	9.3
	Black/African/Caribbean/Black British	1.04	8.61	4.0
	Mixed/Multiple ethnic groups	2.35	4.64	2.9
	Prefer to not answer	0.65	2.32	
Marital status	Married/long-term partner	60.63	62.58	57.83
	Single	25.03	27.15	26.67
	Separated	3.52	2.65	8.50
	Widowed	7.3	5.96	5.62
	Other	3.52	1.66	1.37
Education	Education continued after minimum school leaving age, yes	66.1	71.52	
	Degree, yes	43.68	47.35	
Employment status	Full-time employed	26.6	41.39	34.14
	Part-time employed	8.87	15.56	11.90
	Full-time self-employed	2.09	2.98	5.65
	Part-time self-employed	2.48	2.32	3.82
	Unemployed	4.56	4.3	2.83
	Not in work due to long-term illness or disability	12.65	2.32	4.17
	Taking care of a family member with chronic illness or disability	1.3	0.66	NA‡
	Looking after the home and family	4.43	4.97	4.77
	Retired	35.46	24.83	21.65
	In full or part-time education/ training/ apprenticeship	0.13	0.33	5.64
	Other	1.43	0.33	3.13

COPD indicated chronic obstructive pulmonary disease; NA, not applicable; NHS, National Health Service; QOF, Quality and Outcomes Framework.

∗ These prevalence rates for England only have been obtained from the Quality and Outcomes Framework 2020-21, NHS Digital.

† Statistics for England and Wales from 2021 Census.

‡ This category was not found in the Census 2021 data.

#### Distribution of scores

**Figure 1:**
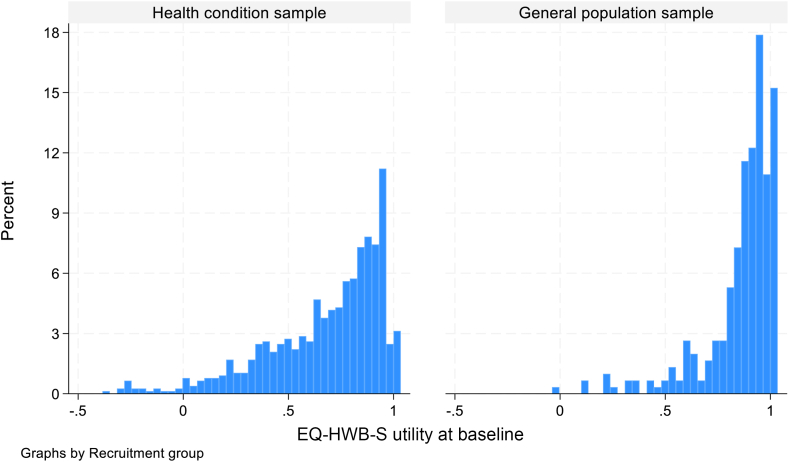
EQ-HWB-S utilities at baseline by groups recruited.

The frequency table for the EQ-HWB items for the 2 samples are presented separately in Appendix Tables 3a and 3b in Supplemental Materials found at https://doi.org/10.1016/j.jval.2025.07.028. Over 60% endorsed the highest level of quality of life for the following items: hearing, self-care, feeling unsafe, and getting around inside and outside. The most impaired level (level 5) is rarely endorsed by any respondents for the first 5 items of the EQ-HWB. The score distributions of the measures for the 2 samples are presented separately in Table 3. The possible scores for EQ-HWB and EQ-HWB-9 range between 25 and 125 and 9 and 45, respectively, in which a higher summative score represents a higher quality of life. The EQ-HWB-S utility scores range from −0.384 to 1. The right-skewed histograms in Figure 1 show that EQ-HWB-S utilities extend across a wider range of scores for the health condition sample than the population sample with 3% and 15% scoring 1 (hence reporting no problems on any of the 9 items) for the health sample and general sample, respectively. Together, these results suggest a low risk of ceiling or floor effect for either of the 2 samples. The sum scores for the EQ-HWB and other measures are presented in histograms in Appendix Figures 1 to 7 in Supplemental Materials found at https://doi.org/10.1016/j.jval.2025.07.028.

**Table 3:** Summary scores at different time points for health condition and general population samples.

Measure and time point	Health condition sample					General population sample				
	*n*	mean	SD	min	max	*n*	mean	SD	min	max
EQ-HWB-S utility at T1	767	0.679	0.276	−0.384 (*n =* 1)	1 (*n =* 24)	302	0.859	0.174	−0.015 (*n =* 1)	1 (*n =* 46)
EQ-HWB-S utility at T2	250	0.707	0.255	−0.384 (*n =* 1)	1 (*n =* 13)	50	0.870	0.157	0.316 (*n =* 1)	1 (*n =* 9)
EQ-HWB level sum score T1	767	55.460	20.034	25 (*n =* 7)	120 (*n =* 1)	302	41.838	14.561	25 (*n =* 9)	98 (*n =* 1)
EQ-HWB level sum score T2	250	53.216	19.013	25 (*n =* 5)	109 (*n =* 1)	50	39.340	13.269	25 (*n =* 3)	88 (*n =* 1)
EQ-HWB-9 level sum score T1	767	20.280	8.127	9 (*n =* 24)	45 (*n =* 1)	302	14.556	5.838	9 (*n =* 46)	36 (*n =* 1)
EQ-HWB-9 level sum score T2	250	19.236	7.751	9 (*n =* 13)	45 (*n =* 1)	50	13.920	5.233	9 (*n =* 9)	33 (*n =* 1)
SWEMWBS total score T1	767	23.044	6.020	7 (*n =* 12)	35 (*n =* 18)	302	25.530	5.636	7 (*n =* 3)	35 (*n =* 17)
SWEMWBS total score T2	250	23.764	5.806	7 (*n =* 1)	35 (*n =* 6)	50	26.480	5.779	11 (*n =* 1)	35 (*n =* 1)
EQ5D-5L utility T1∗	767	0.651	0.266	−0.577 (*n =* 1)	0.987 (*n =* 1)	302	0.858	0.155	0.103 (*n =* 1)	0.986 (*n =* 1)
EQ5D-5L utility T2∗	250	0.678	0.245	−0.577 (*n =* 1)	0.984 (*n =* 1)	50	0.851	0.175	0.225 (*n =* 1)	0.982 (*n =* 1)
ICECAP-A index T1	767	0.721	0.236	−0.001 (*n =* 12)	1 (*n =* 65)	302	0.832	0.183	−0.001 (*n =* 1)	1 (*n =* 49)
PHQ-8 score T1	767	7.420	6.618	0 (118)	24 (*n =* 17)	302	3.338	4.732	0 (*n =* 115)	24 (*n =* 2)
GAD-7 score T1	767	5.900	6.115	0 (*n =* 212)	21 (*n =* 24)	302	2.440	4.033	0 (*n =* 166)	21 (*n =* 2)
UCLA score T1	767	5.068	2.031	3 (*n =* 284)	9 (*n =* 76)	302	4.384	1.700	3 (*n =* 153)	9 (*n =* 10)

∗ Scores are mapped to 3L utility. Therefore min and max should be interpreted with caution.

### Convergent Validity

#### Correlations between EQ-HWB scores and scores from other measures

As shown in Table 4, the summative scores of EQ-HWB and EQ-HWB-9 are highly correlated with a correlation coefficient of 0.98 for the health condition population and 0.97 for the general population. For completeness, the correlations between the various EQ-HWB items are presented in the Appendix Tables 4A and 4B in the Supplemental Materials. All the correlations are higher for the health condition sample compared with the general population for all measures. For the summative scores, the highest correlations are observed for the EQ-HWB measures with EQ-5D-5L and ICECAP-A. For the EQ-HWB-S utilities, the highest correlations for the health condition sample and (general population) are observed with the sum scores of the EQ-HWB measures followed by EQ-5D-5L (r = 0.867 (0.719)) and PHQ-8 (r = −0.8 (−0.715)). The correlations between EQ-HWB-S utilities and ICECAP-A tariff and ASCOT-SC utility scores were 0.706 and 0.571, respectively, for the health condition sample.

**Table 4:** Pearson correlation between EQ-HWB scores and other measures.

Measures	Health condition sample			General population sample		
	EQ-HWB summative score	EQ-HWB-9 summative score	EQ-HWB-S Utility score	EQ-HWB summative score	EQ-HWB-9 summative score	EQ-HWB-S Utility score
EQ-HWB-9 summative score	0.977		−0.952	0.965		−0.949
EQ-HWB-S utility score	−0.938			−0.909		
EQ-5D-5L index∗	0.793	0.783	0.867	0.658	0.675	0.719
SWEMWBS total score∗	0.739	0.721	0.626	0.610	0.544	0.453
ICECAP-A index∗	0.775	0.756	0.706	0.698	0.674	0.644
ASCOT index	0.684†	0.655†	0.571†	NR	NR	NR

None and a little (of the time)	721	−1.57	−1.62	1.58	1.16	1.12	1.47
Some, most and all (of the time)	348						
	Presence of at least one long-term condition						
No	254	−1.13	−1.20	1.40	0.48	1.81	0.66
Yes	815						
Using cutoffs from PHQ-8							
Nonclinical (score 0-9)	781	−2.47	−2.52	2.51	1.48	1.69	1.88
Clinical (score ≥ 10)	288						
Using cutoffs from GAD-7							
Nonclinical (score 0-9)	844	−2.16	−2.18	2.10	1.42	1.56	1.70
Clinical (score ≥ 10)	225						

*Note.* Number of observations are indicated.

∗ These measures were completed by everyone; therefore, *n =* 1069 (all sample); *n =* 767 (health condition); and *n =* 302 (general population).

† *n =* 219 NR, not reported as *n =* 22. Cutoffs for correlation used: ≥0.7, strong evidence; >0.4 to <0.7 moderate correlation; and ≤0.4, weak correlation.

The correlation between arthritis pain visual analog scale is highest with EQ-5D-5L scores and the EQ-HWB measures compared with the rest of the measures. The calculated index of severity for those with rheumatoid arthritis is most highly correlated with the EQ-HWB, EQ-HWB-9 summative scores and PHQ-8.

#### Correlations between EQ-HWB items and items from other measures

##### EQ-HWB and EQ-5D-5L items

Based on the wording of EQ-HWB items and EQ-5D-5L items, 11 instances of similar wording were hypothesized (see Appendix Table 4C in Supplemental Materials found at https://doi.org/10.1016/j.jval.2025.07.028). In all 11 instances, the correlations were higher for the health condition sample compared with the general population sample. Across both samples, only 20 of the 22 item correlations for which hypothesis were set were met. We hypothesized strong correlation between the EQ-5D-5L pain/discomfort item and EQ-HWB item 24 (frequency of discomfort) and EQ-HWB25 (severity of discomfort). However, we observed a moderate correlation of 0.615 and 0.684 for the latter items for the health condition sample. The correlations between the EQ-HWB items measuring pain and discomfort and the EQ-5D-5L pain/discomfort item are higher when using the severity response scale than the frequency scale. For the health condition sample, there was 1 EQ-HWB item that was strongly correlated with each of the mobility, self-care, and usual activities dimensions of the EQ-5D-5L; 3 EQ-HWB items and 2 EQ-HWB items were strongly correlated with the anxious/depressed and the pain/discomfort dimensions, respectively. Exact Spearman correlations can be found in Appendix Tables 4C-4F in Supplemental Materials found at https://doi.org/10.1016/j.jval.2025.07.028.

##### EQ-HWB and SWEMWBS items

The correlations between EQ-HWB and SWEMWBS items are higher in the health condition group. Out of the 12 hypothesized correlations in the health condition group, we expected 6 instances of strong correlations based on the item wordings. However, the correlations between EQ-HWB and SWEMWBS items are at best moderately correlated. Across both samples, only 14 of the 24 item correlations for which hypothesis were set were met. The highest correlations can be observed between the following items for the health condition and (general population) samples: EQ-HWB item 11 “Trouble concentrating or thinking clearly” and SWEMWBS item 5 “Thinking clearly” (r = −0.651 (−0.572)), EQ-HWB item 12 “Anxious” and SWEMWBS item 3 “Feeling relaxed” (r = −0.651 (−0.523)), and EQ-HWB item 19 “Accepted by others” and SWEMWBS item 6 “Feeling close to people” (r = −0.635 (−0.451)).

##### EQ-HWB and ICECAP-A items

The correlations between EQ-HWB and ICECAP-A items are higher in the health condition group. The 13 hypothesized correlations between EQ-HWB and ICECAP-A items lie between −0.258 and −0.684 (see Appendix Table 4E in Supplemental Materials found at https://doi.org/10.1016/j.jval.2025.07.028). For the health condition sample, 7 cases of strong correlations were hypothesized based on the wording; however, they all show moderate correlations. Across both samples, only 8 of the 26 item correlations for which hypothesis were set were met. The highest correlations can be observed for the following items of the health condition and the (general population) samples: EQ-HWB20 “Good about myself” and ICECAP5 “Can have enjoyment and pleasure” (r = −0.684 (−0.501)), EQ-HWB16 “Nothing to look forward to” and ICECAP5 “Can have enjoyment and pleasure” (r = −0.673 (−0.511)), and EQ-HWB21 “Do the things I wanted to do” and ICECAP4 “Can achieve and progress” (r = −0.669 (−0.441)).

##### EQ-HWB and ASCOT items

Eleven hypothesized instances of similar wording between EQ-HWB and ASCOT were identified. Across both samples, only 6 of the 22 item correlations for which hypothesis were set were met. For the health condition group (*n =* 197), the correlations range between 0.298 and 0.605. The correlations for the general population range between 0.091 and 0.719; however, with only 22 individuals in this group, the correlations are to be interpreted with caution.

### Known-Group Validity

The EQ-HWB and EQ-HWB-9 scores are in line with the pre-specified hypotheses have large SES for known-group comparisons based on UCLA,^34^ PHQ-8, GAD-7 cutoffs, presence of a long-term condition, and measure of accomplishing less due to emotional problems (Table 5). Comparing with EQ-5D-5L, ICECAP-A, and SWEMWBS, the EQ-HWB measures have the highest SES for groups defined by emotional problems (defined by GAD-7, PHQ-8, and accomplishing less because of emotional problems). The highest SES are observed for the ICECAP-A scores with the lonely versus not lonely groups as measured by UCLA. EQ-5D-5L have the highest SES for groups identified by virtue of general health (physical). Appendix Table 5 in Supplemental Materials found at https://doi.org/10.1016/j.jval.2025.07.028 shows the SES for all measures for the health condition and general population samples separately. We observe some small differences between the SES of the different EQ-HWB measures but no clear patterns (see Appendix Table 6 in Supplemental Materials found at https://doi.org/10.1016/j.jval.2025.07.028).

**Table 5:** Known-group validity for the EQ-HWB measures (patient and general population samples combined).

Known-groups	*n*	EQ-HWB	Standardized effect size				
			EQ-HWB-9	EQ-HWB-S utility score	SWEMWBS	EQ-5D-5L	ICECAP-A
Using UCLA scores							
Not lonely (score <6)	642	−1.47	−1.49	1.30	−1.18	0.75	1.55
Lonely (score ≥ 6)	427						
Using general health							
Excellent, very good and good	641	1.31	1.27	1.50	0.79	1.54	1.17
Fair and poor	428						
	Accomplished less work and daily activities because of physical health problem						
None and a little (of the time)	632	−1.14	−1.09	1.19	0.61	1.09	0.84
Some, most, and all (of the time)	437						
	Accomplished less work and daily activities because of emotional problems						
None and a little (of the time)	721	−1.57	−1.62	1.58	1.16	1.12	1.47
Some, most and all (of the time)	348						
	Presence of at least 1 long-term condition						
No	254	−1.13	−1.20	1.40	0.48	1.81	0.66
Yes	815						
Using cutoffs from PHQ-8							
Nonclinical (score 0-9)	781	−2.47	−2.52	2.51	1.48	1.69	1.88
Clinical (score ≥ 10)	288						
Using cutoffs from GAD-7							
Nonclinical (score 0-9)	844	−2.16	−2.18	2.10	1.42	1.56	1.70
Clinical (score ≥ 10)	225						

*Note.* To interpret the magnitude of the effect size, the absolute value is used (ignoring the negative sign). The negative sign reflects the opposite ways the measures are scored.

The analyses in the health condition sample show that the EQ-HWB measures can distinguish between the various known groups across all the conditions (see Appendix Table 7 in Supplemental Materials found at https://doi.org/10.1016/j.jval.2025.07.028). Patients with diabetes have lower quality of life as the number of complications (eye, foot, kidney, nerve, gum, and other) increase as measured by all the outcome measures. Patients with arthritis have lower quality of life as the number of joints affected increase, although this relationship is not strictly monotonic. The SES for those with respiratory conditions are highest for EQ-HWB, EQ-HWB-9, and PHQ-8 when comparing those who have at least 1 symptom (asthma, bronchitis, and COPD) with those who are asymptomatic.

### Reliability

#### Test-retest reliability

The ICC for EQ-HWB was 0.900 (*P* < .001) for the health condition sample and 0.931 (*P* < .001) for the general population. The ICC for EQ-HWB-9summative score was 0.876 (*P* < .001) for the health condition sample and 0.914 (*P* < .001) for the general population. The ICC for EQ-HWB-S utility score was 0.866 (*P* < .001) for the health condition sample and 0.933 (*P* < .001) for the general population. Test-retest reliability was higher for the general population sample for all EQ-HWB measures and the SWEMWBS but slightly higher for the health condition sample for EQ-5D-5L. Based on the ICC, the EQ-HWB measures showed slightly better test-retest reliability than either EQ-5D-5L or SWEMWBS, but all measures met the “very good” threshold and direct comparisons of test-retest are challenged by the difference in recall period (see Appendix Table 8 in Supplemental Materials found at https://doi.org/10.1016/j.jval.2025.07.028).

The kappa statistic was also calculated at item level and ranged from 0.335 to 0.562 and from 0.433 to 0.562 for EQ-HWB and EQ-HWB-9, respectively, for the health condition sample. The kappa statistic for the general population sample at item level ranged from 0.288 to 0.684 and from 0.288 to 0.657 for EQ-HWB and EQ-HWB-9, respectively (see Appendix Table 9 in Supplemental Materials found at https://doi.org/10.1016/j.jval.2025.07.028).

## Discussion

This article presents the psychometric properties of the EQ-HWB measures. Results showed that all the response options and the whole measurement range were used. “Very good” test-retest reliability was found at the overall score level for all 3 scoring approaches, but some items showed only “fair” test-retest reliability based on kappa scores. Although some of these items may be expected to show some variability over time even when overall judgement of health has not changed (eg, problems with sleep, feeling exhausted, and pain), for others, the expectation of stability is greater (eg, feeling accepted by others) and the “fair” test-retest findings may be of concern. The seeing, hearing, mobility, and control items, which would also not be expected to show variability over a short time period, showed only “moderate” reliability, as found in previous studies.^17^^,^^21^ Further qualitative research exploring the comprehension of these items may be warranted.

The construct validity of the EQ-HWB measures using sum scores was supported by convergence with fairly similar measures, such as EQ-5D-5L and ICECAP-A. At the item level, correlations were strong between related EQ-HWB and EQ-5D-5L items and, as anticipated, were strongest for the health condition sample for all measures. Correlations to similar items in the ASCOT, ICECAP-A, and SWEMWBS were, in many cases, lower than expected, as also found in for some items in previous studies (eg, ICECAP-A items^15^ and SWEMWBS items^21^). In some cases, this may be because items were linked which were capturing related but not identical concepts. Slight differences in item wording and response options may be driving differences in interpretation (eg, “No control over your day to day life” in EQ-HWB vs “Able to be independent” in ICECAP-A), particularly in cases in which items are framed negatively in the EQ-HWB but positively in the other instrument (eg, “trouble concentrating/thinking clearly” in the EQ-HWB vs “thinking clearly” in the SWEMWBS). Differences could also arise because of differences in the recall period. However, the strongest correlations are seen between EQ-HWB and EQ-5D-5L, which also have different recall periods. The correlations between EQ-HWB and EQ-5D-5L items measuring pain and discomfort are higher using the severity response scale than the ones with a frequency scale, and this is expected because the EQ-5D-5L uses the former. Correlations were higher between the EQ-5D-5L compound pain/discomfort item and the EQ-HWB pain items than the discomfort items, suggesting that the EQ-5D-5L item may be capturing pain more than discomfort. The between instrument correlations, which were all at least moderate, suggest that the measures are capturing related but slightly different constructs. Correlations between the EQ-HWB sum score, EQ-HWB-9 sum score, and EQ-HWB-S utility are always above 0.9, with particularly high correlation between the 25 and 9 item versions when scored as sum score. We also see a higher correlation between EQ-5D-5L and the EQ-HWB-9 utility score than the other EQ-HWB scoring approaches. For the other instruments, the correlations are higher with the EQ-HWB and EQ-HWB-9 sum scored than the EQ-HWB-S utility score. This suggests that greater emphasis is given to the health components that overlap with the EQ-5D-5L in the EQ-HWB-S utility scoring algorithm. The EQ-HWB measures were able to distinguish between the quality of life in the different prespecified known groups. The EQ-HWB measures have large SES for all tested known groups, distinguishing between physical and mental health groups across a range of conditions, as well as between high and low loneliness. The EQ-HWB measures outperform SWEMWBS, ICECAP-A, and EQ-5D-5L when comparing SES across mental health groups and are on a par with the EQ-5D-5L for physical health comparisons. There are minor differences within the EQ-HWB measures, with slightly higher SES for the EQ-HWB long version when distinguishing between groups based on loneliness and whether social care needs are met.

Although the EQ-HWB long version has more granularity, based on the evidence from this research, there are minimal reservations in using the shorter measure for routine data collection, in which the length of the measure is crucial to the patient-reported outcome measures being completed. This work supports other findings that indicate that EQ-5D-5L shows slightly better known-group validity when distinguishing physical health, and EQ-HWB instruments show better performance in distinguishing groups based on mental health.^15^^,^^21^

Although EQ-HWB provides more granularity on certain aspects of quality of life compared with EQ-HWB-9, the psychometric performance of both measures was found to be very similar. The correlation between EQ-HWB and EQ-HWB-9 was above 0.97 for both the health condition and general population samples. In terms of known-group validity, the SES for both measures were similar. The ICC revealed slightly better test-retest reliability for EQ-HWB compared with EQ-HWB-9, but in both instances, the ICC remained above 0.876.

There are several limitations to this study. In the absence of a gold standard in this field, we had to rely on indirect methods to assess construct validity. In this study, the EQ-HWB-9 was embedded in the EQ-HWB and therefore has not been tested as a stand-alone measure. However, we would not expect the results of the validation of the final measure to be different. Third, although the study had a large sample size overall, subgroup analyses by specific self-reported conditions were limited. As a result, in the head-to-head comparison, we could not investigate standardized response means by conditions. Furthermore, instead of using clinical severity measures, we have used crude measures of known-group validity because they were the only ones that could feasibly be collected during the study. The crude groupings need further refinement to be able to build on the evidence presented. Fourth, data collection took place online, and participants were forced to provide a response for every question. As a result, we could not report on feasibility and acceptability of the measures. Fifth, participants in the study did not undergo specific interventions as part of the study. Therefore, the results on reliability need to be interpreted with caution because this is self-reported stability and cannot be verified.

## Conclusions

A UK online survey with a large sample of patients across 6 broadly defined health conditions and a sample of the general population found that the EQ-HWB measures perform well in terms of test-retest reliability (at the overall measure level) and known-group validity, particularly for mental health groups.

## Article and Author Information

**Authorship Confirmation:** All authors certify that they meet the ICMJE criteria for authorship.

**Funding/Support:** This research is funded by the 10.13039/501100000272National Institute for Health Research (NIHR) 10.13039/100024348Policy Research Programme, conducted through the 10.13039/100024348Policy Research Unit in Economic Methods of Evaluation in 10.13039/100017291Health and Social Care Interventions, PR-PRU-1217-20401.

Additional funding for the data collection was obtained from 10.13039/501100000265UK Medical Research Council (grant number 170620) and the 10.13039/501100006419EuroQol Research Foundation.

**Role of the Funder/Sponsor:** The UK 10.13039/501100000265Medical Research Council and the 10.13039/501100006419EuroQol Research Foundation had no role in the design and conduct of the study; collection, management, analysis, and interpretation of the data; preparation, review, or approval of the manuscript; and decision to submit the manuscript for publication.

## Author Disclosures

Author disclosure forms can be accessed below in the Supplemental Material section. The views expressed are those of the author(s) and not necessarily those of the NHS, the National Institute for Health Research, the Department of Health and Social Care or its arm’s length bodies, or other UK government departments. Any errors are the responsibility of the authors. Dr Wailoo is an editor for *Value in Health* and had no role in the peer-review process of this article.

## Supplemental Material

Author Disclosures
