# Supplementary material for: EQ Health and Wellbeing EQ-HWB: A Psychometric Assessment Across 6 Conditions and the General Population in the United Kingdom
Source: Value Health. 2025 Dec;28(12):1857–67. doi: 10.1016/j.jval.2025.07.028 (PMC12711454; doi:10.1016/j.jval.2025.07.028)
Supplement: Author Disclosures [file mmc1.pdf]

Anju Keetharuth

Discloser Identifier: 55026472

Disclosure Purpose: VIH-2025-0202

Employment Information: Currently Employed

Summary of Interests

Company or Organization

Entity	Type	Relevant to this Disclosure
University of Sheffield	Employment Current Employment	Yes
**Title:** Senior Research Fellow		

Additional Questions

1. **Please select which of the following apply to each relationship or activity:**

   a. **Employment** University of Sheffield

   The relationship is in direct support of the work reported in the manuscript anytime from when the work was conceived

2. **I confirm I have disclosed all direct support for the present manuscript (e.g. funding, provision of study materials, medical writing, article processing charges, etc.) There is no time limit for this item.**

   Yes

3. **Please indicate below whether in the past 36 months you have had any of the following interests that are topically related to the work reported in the manuscript.**

   a. **Employment (If you need to add an interest, please scroll to the top of the page, click "add interest" and select “Employment”)**

   No, I have no relevant interests of this type

   b. **Grants or contracts for research (If you need to add an interest, please scroll to the top of the page, click "add interest" and select “Grant/Contract”)**

   No, I have no relevant interests of this type

   c. **Payment for consulting (If you need to add an interest, please scroll to the top of the page, click "add interest" and select “Independent Contractor”)**

   No, I have no relevant interests of this type

   d. **Payments or honoraria for lectures, presentations, speakers bureaus, or educational events (If you need to add an interest, please scroll to the top of the page, click "add interest" and select "Independent Contractor" and include the correct information under "Consultant")**

   No, I have no relevant interests of this type

   e. **Payment for service on an advisory board (If you need to add an interest, please scroll to the top of the page, click "add interest" and select “Independent Contractor,” and choose “Other”)**

   No, I have no relevant interests of this type

f. **Payment for participation Data and safety monitoring board (If you need to add an interest, please scroll to the top of the page, click "add interest" and select “Independent Contractor”)**

No, I have no relevant interests of this type

g. **Expert witness testimony (If you need to add an interest, please scroll to the top of the page, click "add interest" and select “Independent Contractor”)**

No, I have no relevant interests of this type

h. **Royalties from Patents, Trademarks, Copyrights or other intellectual property (If you need to add an interest, please scroll to the top of the page, click "add interest" and select the appropriate interest type)**

No, I have no relevant interests of this type

i. **Patents planned, issued, or pending, whether or not you receive royalties (If you need to add an interest, please scroll to the top of the page, click "add interest" and select “Patents”)**

No, I have no relevant interests of this type

j. **Fiduciary Officer or Other Board Membership (If you need to add an interest, please scroll to the top of the page, click "add interest" and select “Fiduciary Officer”)**

No, I have no relevant interests of this type

k. **Stock or stock options (If you need to add an interest, please scroll to the top of the page, click "add interest" and select the appropriate interest type)**

No, I have no relevant interests of this type

l. **Support for attending meetings or other travel (If you need to add an interest, please scroll to the top of the page, click "add interest" and select “Travel”)**

No, I have no relevant interests of this type

4. **Was any individual paid to provide professional writing assistance with this manuscript?**

No.

5. **Have you or your institution received equipment, materials, drugs, or services in direct support of the work in the manuscript (without time limit) not disclosed above?**

No.

6. **In the past 36 months, have you received equipment, materials, drugs, medical writing, gifts or other services from for-profit or not-for-profit third parties whose interests may be affected by the content of the manuscript not disclosed above?**

No.

7. **Are there other financial or non-financial interests that readers could perceive to have influenced, or that give the appearance of potentially influencing, what you wrote in the submitted work not disclosed above.**

No.

Certification

I certify that I have answered every question and the information provided in this disclosure is complete and accurate.

Clara Mukuria

Discloser Identifier: 55029620

Disclosure Purpose: VIH-2025-0202

Employment Information: Currently Employed

Summary of Interests

Company or Organization

Entity	Type	Relevant to this Disclosure
CM HE Consulting	Employment Current Employment	Yes
**Title:** Senior Researcher		
EuroQol Research Foundation	Grant / Contract	Yes
EuroQol Research Foundation	Other	Yes
**Category:** Other		

Additional Questions

1. **Please select which of the following apply to each relationship or activity:**
   a. **Other Professional Activities - Other** EuroQol Research Foundation

      The relationship is in direct support of the work reported in the manuscript anytime from when the work was conceived

   b. **Employment** CM HE Consulting

      The relationship is outside the work reported in the manuscript but topically related and within the past 36 months

   c. **Grant / Contract** EuroQol Research Foundation

      The relationship is in direct support of the work reported in the manuscript anytime from when the work was conceived

2. **I confirm I have disclosed all direct support for the present manuscript (e.g. funding, provision of study materials, medical writing, article processing charges, etc.) There is no time limit for this item.**

   Yes

3. **Please indicate below whether in the past 36 months you have had any of the following interests that are topically related to the work reported in the manuscript.**
   a. **Employment (If you need to add an interest, please scroll to the top of the page, click "add interest" and select "Employment")**

      Yes, as disclosed above

   b. **Grants or contracts for research (If you need to add an interest, please scroll to the top of the page, click "add interest" and select "Grant/Contract")**

      Yes, as disclosed above

   c. **Payment for consulting (If you need to add an interest, please scroll to the top of the page, click "add interest" and select "Independent Contractor")**

Yes, as disclosed above

d. **Payments or honoraria for lectures, presentations, speakers bureaus, or educational events (If you need to add an interest, please scroll to the top of the page, click "add interest" and select "Independent Contractor" and include the correct information under "Consultant")**

No, I have no relevant interests of this type

e. **Payment for service on an advisory board (If you need to add an interest, please scroll to the top of the page, click "add interest" and select “Independent Contractor,” and choose “Other”)**

No, I have no relevant interests of this type

f. **Payment for participation Data and safety monitoring board (If you need to add an interest, please scroll to the top of the page, click "add interest" and select “Independent Contractor”)**

No, I have no relevant interests of this type

g. **Expert witness testimony (If you need to add an interest, please scroll to the top of the page, click "add interest" and select “Independent Contractor”)**

No, I have no relevant interests of this type

h. **Royalties from Patents, Trademarks, Copyrights or other intellectual property (If you need to add an interest, please scroll to the top of the page, click "add interest" and select the appropriate interest type)**

No, I have no relevant interests of this type

i. **Patents planned, issued, or pending, whether or not you receive royalties (If you need to add an interest, please scroll to the top of the page, click "add interest" and select “Patents”)**

No, I have no relevant interests of this type

j. **Fiduciary Officer or Other Board Membership (If you need to add an interest, please scroll to the top of the page, click "add interest" and select “Fiduciary Officer”)**

No, I have no relevant interests of this type

k. **Stock or stock options (If you need to add an interest, please scroll to the top of the page, click "add interest" and select the appropriate interest type)**

No, I have no relevant interests of this type

l. **Support for attending meetings or other travel (If you need to add an interest, please scroll to the top of the page, click "add interest" and select “Travel”)**

No, I have no relevant interests of this type

4. **Was any individual paid to provide professional writing assistance with this manuscript?**

No.

5. **Have you or your institution received equipment, materials, drugs, or services in direct support of the work in the manuscript (without time limit) not disclosed above?**

No.

6. **In the past 36 months, have you received equipment, materials, drugs, medical writing, gifts or other services from for-profit or not-for-profit third parties whose interests may be affected by the content of the manuscript not disclosed above?**

No.

7. **Are there other financial or non-financial interests that readers could perceive to have influenced, or that give the appearance of potentially influencing, what you wrote in the submitted work not disclosed above.**

Yes.

a. **Please describe the interest or activity.**

Member of the EuroQol Research Association and the EQ-HWB Working Group

Certification

I certify that I have answered every question and the information provided in this disclosure is complete and accurate.

Convey
Global Disclosure System
AAMC

Tessa Peasgood

**Discloser Identifier:**	112453634	**Disclosure Purpose:**	VIH-2025-0202	**Employment Information:**	Currently Employed

Summary of Interests

Company or Organization

Entity	Type	Relevant to this Disclosure
EuroQol Research Foundation	Other Current Employment	Yes
**Category:** Other		
University of Sheffield	Employment Current Employment	
**Title:** Senior Lecturer		

Additional Questions

1. **Please select which of the following apply to each relationship or activity:**
   a. **Other Professional Activities - Other** EuroQol Research Foundation

      The relationship is in direct support of the work reported in the manuscript anytime from when the work was conceived

   b. **Employment** University of Sheffield

      The relationship is outside the work reported in the manuscript but topically related and within the past 36 months

2. **I confirm I have disclosed all direct support for the present manuscript (e.g. funding, provision of study materials, medical writing, article processing charges, etc.) There is no time limit for this item.**

   Yes

3. **Please indicate below whether in the past 36 months you have had any of the following interests that are topically related to the work reported in the manuscript.**
   a. **Employment (If you need to add an interest, please scroll to the top of the page, click "add interest" and select "Employment")**

      Yes, as disclosed above

   b. **Grants or contracts for research (If you need to add an interest, please scroll to the top of the page, click "add interest" and select "Grant/Contract")**

      Yes, as disclosed above

   c. **Payment for consulting (If you need to add an interest, please scroll to the top of the page, click "add interest" and select "Independent Contractor")**

      No, I have no relevant interests of this type

   d. **Payments or honoraria for lectures, presentations, speakers bureaus, or educational events (If you need to add an interest, please scroll to the top of the page, click "add interest" and select "Independent Contractor" and include the**

correct information under "Consultant")**

Yes, as disclosed above

e. **Payment for service on an advisory board (If you need to add an interest, please scroll to the top of the page, click "add interest" and select “Independent Contractor,” and choose “Other”)**

No, I have no relevant interests of this type

f. **Payment for participation Data and safety monitoring board (If you need to add an interest, please scroll to the top of the page, click "add interest" and select “Independent Contractor”)**

No, I have no relevant interests of this type

g. **Expert witness testimony (If you need to add an interest, please scroll to the top of the page, click "add interest" and select “Independent Contractor”)**

No, I have no relevant interests of this type

h. **Royalties from Patents, Trademarks, Copyrights or other intellectual property (If you need to add an interest, please scroll to the top of the page, click "add interest" and select the appropriate interest type)**

No, I have no relevant interests of this type

i. **Patents planned, issued, or pending, whether or not you receive royalties (If you need to add an interest, please scroll to the top of the page, click "add interest" and select “Patents”)**

No, I have no relevant interests of this type

j. **Fiduciary Officer or Other Board Membership (If you need to add an interest, please scroll to the top of the page, click "add interest" and select “Fiduciary Officer”)**

No, I have no relevant interests of this type

k. **Stock or stock options (If you need to add an interest, please scroll to the top of the page, click "add interest" and select the appropriate interest type)**

No, I have no relevant interests of this type

l. **Support for attending meetings or other travel (If you need to add an interest, please scroll to the top of the page, click "add interest" and select “Travel”)**

Yes, as disclosed above

4. **Was any individual paid to provide professional writing assistance with this manuscript?**

   No.

5. **Have you or your institution received equipment, materials, drugs, or services in direct support of the work in the manuscript (without time limit) not disclosed above?**

   No.

6. **In the past 36 months, have you received equipment, materials, drugs, medical writing, gifts or other services from for-profit or not-for-profit third parties whose interests may be affected by the content of the manuscript not disclosed above?**

   No.

7. **Are there other financial or non-financial interests that readers could perceive to have influenced, or that give the appearance of potentially influencing, what you wrote in the submitted work not disclosed above.**

   No.

Certification

I certify that I have answered every question and the information provided in this disclosure is complete and accurate.

Allan Wailoo

Discloser Identifier: 55105698

Disclosure Purpose: VIH-2025-0202

Employment Information: Currently Employed

Summary of Interests

Company or Organization

Entity	Type	Relevant to this Disclosure
National Institute for Health Research	Grant / Contract	Yes
University of Sheffield	Employment Current Employment	
**Title:** Professor of Health Economics		

Additional Questions

1. **Please select which of the following apply to each relationship or activity:**
   a. **Employment** University of Sheffield

      The relationship is in direct support of the work reported in the manuscript anytime from when the work was conceived

   b. **Grant / Contract** National Institute for Health Research

      The relationship is in direct support of the work reported in the manuscript anytime from when the work was conceived

2. **I confirm I have disclosed all direct support for the present manuscript (e.g. funding, provision of study materials, medical writing, article processing charges, etc.) There is no time limit for this item.**

   Yes

3. **Please indicate below whether in the past 36 months you have had any of the following interests that are topically related to the work reported in the manuscript.**
   a. **Employment (If you need to add an interest, please scroll to the top of the page, click "add interest" and select “Employment”)**

      No, I have no relevant interests of this type

   b. **Grants or contracts for research (If you need to add an interest, please scroll to the top of the page, click "add interest" and select “Grant/Contract”)**

      No, I have no relevant interests of this type

   c. **Payment for consulting (If you need to add an interest, please scroll to the top of the page, click "add interest" and select “Independent Contractor”)**

      No, I have no relevant interests of this type

   d. **Payments or honoraria for lectures, presentations, speakers bureaus, or educational events (If you need to add an interest, please scroll to the top of the page, click "add interest" and select "Independent Contractor" and include the correct information under "Consultant")**

      No, I have no relevant interests of this type

e. **Payment for service on an advisory board (If you need to add an interest, please scroll to the top of the page, click "add interest" and select “Independent Contractor,” and choose “Other”)**

No, I have no relevant interests of this type

f. **Payment for participation Data and safety monitoring board (If you need to add an interest, please scroll to the top of the page, click "add interest" and select “Independent Contractor”)**

No, I have no relevant interests of this type

g. **Expert witness testimony (If you need to add an interest, please scroll to the top of the page, click "add interest" and select “Independent Contractor”)**

No, I have no relevant interests of this type

h. **Royalties from Patents, Trademarks, Copyrights or other intellectual property (If you need to add an interest, please scroll to the top of the page, click "add interest" and select the appropriate interest type)**

No, I have no relevant interests of this type

i. **Patents planned, issued, or pending, whether or not you receive royalties (If you need to add an interest, please scroll to the top of the page, click "add interest" and select “Patents”)**

No, I have no relevant interests of this type

j. **Fiduciary Officer or Other Board Membership (If you need to add an interest, please scroll to the top of the page, click "add interest" and select “Fiduciary Officer”)**

No, I have no relevant interests of this type

k. **Stock or stock options (If you need to add an interest, please scroll to the top of the page, click "add interest" and select the appropriate interest type)**

No, I have no relevant interests of this type

l. **Support for attending meetings or other travel (If you need to add an interest, please scroll to the top of the page, click "add interest" and select “Travel”)**

No, I have no relevant interests of this type

4. **Was any individual paid to provide professional writing assistance with this manuscript?**

   No.

5. **Have you or your institution received equipment, materials, drugs, or services in direct support of the work in the manuscript (without time limit) not disclosed above?**

   No.

6. **In the past 36 months, have you received equipment, materials, drugs, medical writing, gifts or other services from for-profit or not-for-profit third parties whose interests may be affected by the content of the manuscript not disclosed above?**

   No.

7. **Are there other financial or non-financial interests that readers could perceive to have influenced, or that give the appearance of potentially influencing, what you wrote in the submitted work not disclosed above.**

   No.

Certification

I certify that I have answered every question and the information provided in this disclosure is complete and accurate.
