# Supplementary material for: EQ Health and Wellbeing EQ-HWB: A Psychometric Assessment Across 6 Conditions and the General Population in the United Kingdom
Source: Value Health. 2025 Dec;28(12):1857–67. doi: 10.1016/j.jval.2025.07.028 (PMC12711454; doi:10.1016/j.jval.2025.07.028)
Supplement: Supplemental Material [file mmc2.docx]

**Appendix 1 Survey outline and questions**

** Actual formatting and question layout was amended based on survey company data collection interface

** Copyrighted questionnaires are only referred to by name

** Note that survey began with an information sheet describing the study and a consent form neither of which are included here.

*****************

***Thank you for agreeing to take part. Please answer all the questions. Some questions may seem very similar – please answer them all as this will allow us to compare different measures.***

This section covers questions that are about you. Please answer each question, please give an answer by ticking **(**✓**)** where questions have options.

How old are you? ________________ years

What is your gender?

| Male | 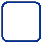 |
| --- | --- |
| Female | 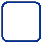 |
| Transgender male | 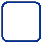 |
| Transgender female | 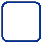 |
| Non-binary | 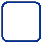 |
| Prefer not to say | 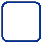 |

What is your ethnic group?

| White British | 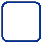 |
| --- | --- |
| White non-British | 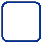 |
| Asian / Asian British | 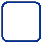 |
| Black / African / Caribbean / Black British | 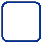 |
| Mixed / Multiple ethnic groups | 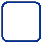 |
| Other ethnic group | 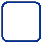 |
| Prefer not to say | 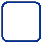 |

What is your current marital status?

| Married / long-term partner | 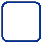 |
| --- | --- |
| Single | 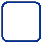 |
| Separated | 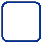 |
| Widowed | 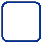 |
| Other | 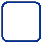 |

Has your marital status changed within the last 12 months? [Yes/No]

Who do you live with?

| I live alone | 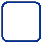 |
| --- | --- |
| I live in a shared house with immediate/ extended family | 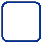 |
| I live in a shared house with friends | 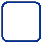 |
| I live in a shared house with other people (not friends or family) | 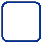 |
| I live in sheltered or residential housing | 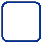 |
| Prefer not to say | 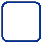 |

For those who say they live with others

How many more people do you live with?

| 1 extra person | 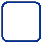 |
| --- | --- |
| 2 extra people | 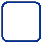 |
| 3 extra people | 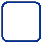 |
| 4 or more extra people | 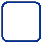 |
| Prefer not to say | 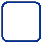 |

Do you have any children?

| Yes (aged below 18) | 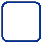 |
| --- | --- |
| Yes (aged 18 and over) | 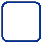 |
| No | 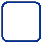 |

Did your education continue after the minimum school leaving age? [Yes/No]

Do you have a degree or equivalent professional qualification? [Yes/No]

What is your employment status? Employment is about your main daily activity. If you are currently on maternity/ parental/ sick or furlough leave, you count as being employed.

| Full-time employed | 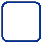 |
| --- | --- |
| Part-time employed | 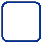 |
| Full-time self-employed | 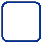 |
| Part-time self-employed | 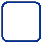 |
| Unemployed (looking for work) | 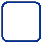 |
| Not in work due to long-term illness or disability | 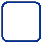 |
| Taking care of a family member with chronic illness or disability | 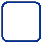 |
| Looking after the home and family | 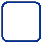 |
| Retired | 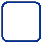 |
| In full or part time education/ training/ apprenticeship | 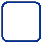 |
| Other | 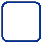 |

For those employed/self-employed

Are you currently on:

| Furlough leave | 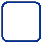 |
| --- | --- |
| Maternity / parental leave | 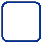 |
| Sick leave | 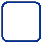 |
| Self-employment income support | 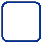 |
| None of the above | 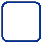 |

For those who are employed but not on any leave i.e. if ‘none of the above’

Are you currently:

| Working in your regular place of work | 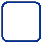 |
| --- | --- |
| Working in a different location [e.g. working from home instead of the office] | 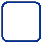 |

Has your employment status changed within the last 12 months? [Yes/No]

If yes

What was your previous employment status? If you were previously on maternity/ parental/ sick or furlough leave, you count as being employed.

| Full-time employed | 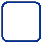 |
| --- | --- |
| Part-time employed | 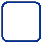 |
| Full-time self-employed | 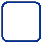 |
| Part-time self-employed | 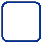 |
| Unemployed (looking for work) | 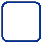 |
| Not in work due to long-term illness or disability | 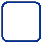 |
| Taking care of a family member with chronic illness or disability | 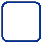 |
| Looking after the home and family | 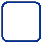 |
| Retired | 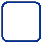 |
| In full or part-time education/ training/ apprenticeship | 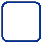 |
| Other | 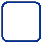 |

What is your **household disposable income** (i.e. the money that is left from your pay or income after deducting tax, NI contributions and after you have paid for your housing costs (mortgage/rent or similar housing costs)?

|  | Monthly (£) | or | Weekly (£) |
| --- | --- | --- | --- |
| Please enter an amount |  | or |  |
| I don’t know | 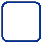 |  |  |
| Prefer not to say | 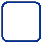 |  |  |

Has your income changed within the last 12 months?

| Yes – it has increased | 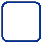 |
| --- | --- |
| Yes – it has decreased | 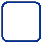 |
| No | 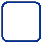 |

Thinking about your **home**, would you say it is in a reasonable state of repair, with reasonable facilities (cooking/washing) and that it provides reasonable warmth

| All true | 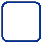 |
| --- | --- |
| Partly true | 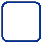 |
| Not true | 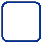 |
| Prefer not to say | 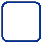 |

This section asks about your overall life. For each question please give an answer by ticking **(**✓**)**.

Overall, how satisfied are you with your life nowadays?

0 1 2 3 4 5 6 7 8 9 10

Not at all Completely

Overall, to what extent do you feel the things you do in your life are worthwhile?

0 1 2 3 4 5 6 7 8 9 10

Not at all Completely

Overall, how happy were you feeling yesterday?

0 1 2 3 4 5 6 7 8 9 10

Not at all Completely

Overall, how anxious were you feeling yesterday?

(*10 would mean you are completely anxious)*

0 1 2 3 4 5 6 7 8 9 10

Not at all Completely

** UCLA 3 Item Loneliness scale

Thinking about the area in which you live, how often are you concerned with the **safety of the neighbourhood**?

| Hardly ever | 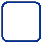 |
| --- | --- |
| Some of the time | 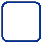 |
| All of the time | 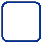 |
| Prefer not to say | 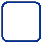 |

For each question please give an answer by ticking **(**✓**)**.

In general, would you say your health is:

Excellent Very good Good Fair Poor

In the past 4 weeks, have you **accomplished less** than you would like with your work or other regular daily activities as a result of your **physical health**?

| None of the time | 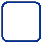 |
| --- | --- |
| A little of the time | 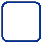 |
| Some of the time | 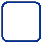 |
| Most of the time | 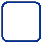 |
| All of the time | 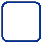 |

In the past 4 weeks, have you **accomplished less** than you would like with your work or other regular daily activities as a result of your **emotional problems** (such as feeling depressed or anxious)?

| None of the time | 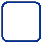 |
| --- | --- |
| A little of the time | 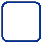 |
| Some of the time | 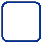 |
| Most of the time | 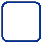 |
| All of the time | 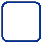 |

Are you worried about getting COVID-19?

| Yes – I am very worried | 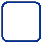 |
| --- | --- |
| Yes – I am moderately worried | 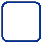 |
| Yes – I slightly worried | 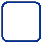 |
| No – I am not worried | 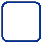 |
| No – I have already had it | 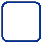 |

Are you currently ill with COVID-19:

| Yes – I have **undiagnosed mild** symptoms | 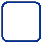 |
| --- | --- |
| Yes – I have **undiagnosed moderate** symptoms | 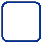 |
| Yes – I have **diagnosed mild** symptoms (not needing hospitalisation) | 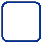 |
| Yes – I have **diagnosed moderate** symptoms (not needing hospitalisation) | 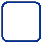 |
| None of the above | 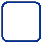 |

Have you been previously ill with COVID-19:

| Yes – I had **undiagnosed mild** symptoms | 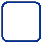 |
| --- | --- |
| Yes – I had **undiagnosed moderate** symptoms | 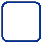 |
| Yes – I had **diagnosed mild or moderate** symptoms (not needing hospitalisation) | 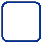 |
| Yes – I had **diagnosed moderate to severe** symptoms (needing hospitalisation) | 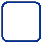 |
| None of the above | 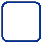 |

Are you currently experiencing or have you had COVID-19 symptoms (undiagnosed or diagnosed) that have had an impact on you for longer than 4 weeks?

| Yes | 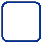 | No |  |
| --- | --- | --- | --- |

Is anyone else living with you currently ill with COVID-19:

| Yes – they have **undiagnosed mild** symptoms |  |
| --- | --- |
| Yes – they have **undiagnosed moderate** symptoms |  |
| Yes – they have **diagnosed mild** symptoms (not needing hospitalisation) |  |
| Yes – they have **diagnosed moderate** symptoms (not needing hospitalisation) |  |
| Yes – they have **diagnosed moderate to severe** symptoms (needing hospitalisation) |  |
| None of the above |  |

Do you have any long-standing physical or mental health impairment, illness or disability which has been diagnosed by a doctor? By 'long-standing' we mean anything that has troubled you over a period of at least 12 months or that is likely to trouble you over a period of at least 12 months.

| Yes |  | No |  |
| --- | --- | --- | --- |

If yes to Q31 long-standing physical or mental health impairment

|  | Please tick **all** that apply: | Please select **one** condition with biggest impact on you |
| --- | --- | --- |
| Asthma [If yes ask asthma que] |  |  |
| Anxiety disorder [If yes ask Mental health que] |  |  |
| Arthritis [If yes ask Arthritis Questions] |  |  |
| Cancer or malignancy |  |  |
| Chronic bronchitis [If yes ask asthma que] |  |  |
| Chronic fatigue |  |  |
| Clinical depression [If yes Mental health que] |  |  |
| Diabetes |  |  |
| Emphysema/ COPD [If yes ask asthma que] |  |  |
| Epilepsy |  |  |
| Heart condition |  |  |
| High blood pressure |  |  |
| Irritable bowel condition |  |  |
| Liver condition |  |  |
| Migraine/chronic headaches |  |  |
| Stroke |  |  |
| Thyroid problems (over-active or underactive) |  |  |
| Other physical health condition |  |  |
| Other mental health condition |  |  |

If other physical or mental health condition, please tell us the condition:

Thinking of the health condition that you selected as having the biggest impact on you, please choose the response below that best describes the **severity of your condition** over the past week:

| No problems/symptoms |  |
| --- | --- |
| Mild problems/symptoms |  |
| Moderate problems/symptoms |  |
| Severe problems/symptoms |  |

How difficult is it for you to look after yourself (wash, use the toilet, dress yourself, eat or care for your appearance)?

| These things are very easy for me to do |  |
| --- | --- |
| I find some of these things difficult, but I manage to do them on my own |  |
| Many of these things are difficult, and I need help to do them |  |
| I cannot do these things by myself at all. |  |

Do you receive any practical help or care on a regular basis because you cannot do things by yourself?

**Please tick all that apply**

| Yes from someone living with me (e.g. partner) |  |
| --- | --- |
| Yes from someone who does not live with me (e.g. family or friends) |  |
| Yes from someone who is paid for by social services (e.g. a carer) |  |
| Yes from someone who you pay for with your own money |  |
| Yes from someone who is paid for by your family |  |
| Yes from a volunteer from a charity |  |
| No – but I need help |  |
| No – I do not need help |  |

IF **yes – Q33**  filter to social care questions (at end).

We want to know if you regularly provide help to anyone who is sick, disabled or elderly either living with you or not living with you but not as part of your job. Is there anyone like this who you look after?

| Yes |  | No |  |
| --- | --- | --- | --- |

IF **yes Q34 –** filter to carer-related questions (at end)**.**

**** In random order: EQ-HWB, Short WEWMBS, ICECAP-A and EQ-5D-5L**

**For those who are recruited or say they have anxiety or depression:**

Length of time with condition [Years and months]

Are you taking medication to manage your anxiety/depression?

Yes – prescribed by a doctor

Yes – over the counter medication (not prescribed)

No

Are you currently receiving any psychological therapy or counselling for your anxiety/depression? [Yes/No]

Are you on a waiting list to receive psychological therapy or counselling for you anxiety/depression? [Yes/No]

Has your anxiety/depression been affected by the COVID-19 pandemic:

| Yes – it has increased my symptoms **slightly** |  |
| --- | --- |
| Yes – it has increased my symptoms **a lot** |  |
| No – it has had **no impact** on my symptoms |  |

**** Include Patient Health Questionnaire -8 (PHQ-8)**

**** Include GAD 7**

**For those who are recruited or say they have diabetes:**

Which type of diabetes do you have?

Type 1

Type 2

I don’t know

Length of time with diabetes? [Years and months]

Are you currently using insulin for your diabetes? [Yes/No]

In the past 6 months have you had an episode of hypoglycaemia that required an ambulance/paramedic call out or a visit to the emergency department? [Yes/No]

In the past 6 months have you had an admission to hospital because of your diabetes? [Yes/No]

Do you have any of these complications because of your diabetes? (Tick all that apply)

- Eye problems (diabetic retinopathy)
- Foot problems (e.g. wounds that do not heal)
- Kidney problems (diabetic nephropathy)
- Nerve damage
- Gum disease and other mouth problems
- Other diabetic complications (Please state):

Has your diabetes been affected by the COVID-19 pandemic:

| Yes – it has made it **slightly more** difficult to manage |  |
| --- | --- |
| Yes – it has made it **a lot more** difficult to manage |  |
| No – it has had **no impact** |  |

**For those with arthritis:**

Which type of arthritis do you have?

Rheumatoid arthritis

Osteoarthritis

Other

I don’t know

Length of time with arthritis? [Years and months]

Where does your arthritis affect you? (Tick all that apply)

| Back |  |
| --- | --- |
| Feet |  |
| Hands and wrists |  |
| Hips |  |
| Knees |  |
| Neck |  |
| Other area |  |

Pain VAS 0 to 100 [Horizontal Scale with 0 as no pain and 100 as worst pain]

Are you currently taking any medication for your arthritis?

Yes – prescribed by a doctor

Yes – over the counter medication (not prescribed)

No

If yes, how many different kinds of medication are you taking e.g. if you are only taking one type of painkiller select ‘1’, if you are taking two different painkillers and a one arthritis drug select ‘3’? [options 1,2,3,4,5 and 6 or more]

Do you currently have other therapy such as physiotherapy to manage your arthritis? [Yes/No]

*For Osteoarthritis participants*

Are you currently on a waiting list for surgery due to your arthritis? [Yes/No]

*For RA participants*

How active was your arthritis the last **month**

0 1 2 3 4 5 6 7 8 9 10

Completely Extremely

Inactive Active

How active is your arthritis **today** with respect to joint tenderness and swelling?

0 1 2 3 4 5 6 7 8 9 10

Completely Extremely

Inactive Active

How severe us your arthritis pain **today**?

0 1 2 3 4 5 6 7 8 9 10

No Unbearable

Pain Pain

How would you describe your general health **today** *(0 is excellent and 10 is extremely bad)*

0 1 2 3 4 5 6 7 8 9 10

Excellent Extremely

Bad

Did you experience joint stiffness on awaking **yesterday** morning? If yes how long was this stiffness?

0 1 2 3 4 5 6 7 8 9 10

No Stiffness

Stiffness the whole day

Has your arthritis been affected by the COVID-19 pandemic:

| Yes – it has made it **slightly more** difficult to manage |  |
| --- | --- |
| Yes – it has made it **a lot more** difficult to manage |  |
| No – it has had **no impact** |  |

**For those who are recruited or say they have chronic bronchitis or asthma or COPD:**

Length of time with asthma [chronic bronchitis/COPD]? [Yes/No]

Are you currently using medication for your asthma [chronic bronchitis/COPD]? [Yes/No]

In the past **6 months** have you had an episode because of your asthma [chronic bronchitis/COPD] that required an ambulance/paramedic call out or a visit to the emergency department? [Yes/No]

In the past **6 months** have you had an admission to hospital because of your asthma [chronic bronchitis/COPD]? [Yes/No]

In the past **week** have you experienced any of the symptoms below because of your asthma [chronic bronchitis/COPD]:

|  | **None of the time** | **Occasional (2-3 days)** | **Most days** | **Every day** |
| --- | --- | --- | --- | --- |
| Coughing, wheezing or shortness of breath when you first wake up |  |  |  |  |
| Daytime coughing |  |  |  |  |
| Daytime wheezing or tightness |  |  |  |  |
| Daytime shortness of breath |  |  |  |  |
| Night-time coughing, wheezing or shortness of breath |  |  |  |  |

Has your chronic bronchitis/asthma/COPD been affected by the COVID-19 pandemic:

| Yes – it has made it **slightly more** difficult to manage |  |
| --- | --- |
| Yes – it has made it **a lot more** difficult to manage |  |
| No – it has had **no impact** |  |

**For those who are informal carers (answer yes to Q34 plus recruited):**

**[Questions drawn from the Social Services Carer Survey which uses the ASCOT Carer alongside a number of other questions]**

If you look after a family member, partner or friend in need of support or services because of their age, physical or learning disability or illness, including mental illness, we would like to find out more about the person you care for and how you find caring.

| **About the person you care for and the care you provide**  The questions in this section ask about the person you care for, by which we mean the person you look after or help. |
| --- |

| If you care for more than one person, please answer **only** in relation to the person you spend the most time helping. If you spend an equal amount of time caring for two or more people, please answer in relation to the person who lives with you. If you live with two or more people that you spend an equal amount of time caring for, please choose **one** person to answer about. |
| --- |

How old is the person you care for? **_____________years**

(If you don’t know the exact age, please give an approximate one)

Does the person you care for have....?

***Please tick [✓] all that apply***

|  |  | A learning disability or difficulty |
| --- | --- | --- |
|  |  |  |
|  |  | A physical disability |
|  |  |  |
|  |  | Sight or hearing loss |
|  |  |  |
|  |  | A mental health problem |
|  |  |  |
|  |  | Dementia |
|  |  |  |
|  |  | Problems connected to ageing |
|  |  |  |
|  |  | Long-standing illness |
|  |  |  |
|  |  | Alcohol or drug dependency |
|  |  |  |
|  |  | Other |

Where does the person you care for usually live?

***Please tick [✓] one box***

|  |  | With me |
| --- | --- | --- |
|  |  |  |
|  |  | Somewhere else |

Does the person you care for receive **any other support or services from anyone else?**

| Yes from other family or friends |  |
| --- | --- |
| Yes from social services |  |
| Yes from services paid for privately |  |
| Yes from a volunteer from a charity |  |
| No |  |

About how long have you been looking after or helping the person you care for?

**Years Months**

About how long do you spend **each week** looking after or helping the person you care for?

***Please tick [✓] one box***

|  |  | 0-9 hours per week |
| --- | --- | --- |
|  |  |  |
|  |  | 10-19 hours per week |
|  |  |  |
|  |  | 20-34 hours per week |
|  |  |  |
|  |  | 35-49 hours per week |
|  |  |  |
|  |  | 50-74 hours per week |
|  |  |  |
|  |  | 75-99 hours per week |
|  |  |  |
|  |  | 100 or more hours per week |

What kinds of things do you usually do for the person you care for?

***Please tick [✓] all that apply***

|  |  | Personal care? |
| --- | --- | --- |
|  |  | (Things like dressing, bathing, washing, shaving, cutting nails, feeding, using the toilet) |
|  |  |  |
|  |  | Physical help? |
|  |  | (Such as helping with walking, getting up and down stairs, getting into and out of bed) |
|  |  |  |
|  |  | Helping with dealing with care services and benefits? |
|  |  | (Things like making appointments and phone calls, filling in forms) |
|  |  |  |
|  |  | Helping with paperwork or financial matters? |
|  |  | (Such as writing letters, sending cards, filling in forms, dealing with bills, banking) |
|  |  |  |
|  |  | Other practical help? |
|  |  | (Things like preparing meals, doing his/her shopping, laundry, housework, gardening, decorating, household repairs, taking to doctor’s or hospital) |
|  |  |  |
|  |  | Keeping him/her company? |
|  |  | (Things like visiting, sitting with, reading to, talking to, playing cards or games) |
|  |  |  |
|  |  | Taking him/her out? |
|  |  | (Such as taking out for a walk or drive, taking to see friends or relatives) |
|  |  |  |
|  |  | Giving medicines? |
|  |  | (Things like making sure he/she takes pills, giving injections, changing dressings) |
|  |  |  |
|  |  | Keeping an eye on him/her to see he/she is all right? |
|  |  |  |
|  |  |  |
|  |  | Giving emotional support? |
|  |  |  |
|  |  |  |
|  |  | Other help? |

Thinking about the **other people you have caring responsibilities for**, which of the following best describes your current situation? Please exclude the person you spend most time helping.

***Please tick [✓] one box***

|  | I don’t have caring responsibilities for anyone else |
| --- | --- |

|  | I always have enough time to care for them |
| --- | --- |
|  |  |
|  | I sometimes have enough time to care for them |
|  |  |
|  | I never have enough time to care for them |

**** Include ASCOT Carer 4 level self-completion SCT4 v1.1 (with IP)**

**** Include Carer Experience Scale (CES)**

In the last **month**, has your health been affected by your caring role in any of the ways listed below?

***Please tick [✓] all that apply***

|  | Feeling tired |
| --- | --- |
|  |  |
|  | Feeling depressed |
|  |  |
|  | Loss of appetite |
|  |  |
|  | Disturbed sleep |
|  |  |
|  | General feeling of stress |
|  |  |
|  | Physical strain (e.g. back) |
|  |  |
|  | Short tempered/ irritable |
|  |  |
|  | Had to see own GP |
|  |  |
|  | Developed my own health conditions |
|  |  |
|  | Made an existing condition worse |
|  |  |
|  | Other |
|  |  |
|  | No, none of these |

Has your caring role been affected by the COVID-19 pandemic:

| Yes – it has made it **slightly more** difficult to manage |  |
| --- | --- |
| Yes – it has made it **a lot more** difficult to manage |  |
| No – it has had **no impact** |  |

**For those who identify that they receive social care support (answer yes to question 33):**

What kinds of things do you usually get support for?

***Please tick [✓] all that apply***

|  |  | Personal care? |
| --- | --- | --- |
|  |  | (Things like dressing, bathing, washing, shaving, cutting nails, feeding, using the toilet) |
|  |  |  |
|  |  | Physical help? |
|  |  | (Such as help with walking, getting up and down stairs, getting into and out of bed) |
|  |  |  |
|  |  | Help with dealing with care services and benefits? |
|  |  | (Things like making appointments and phone calls, filling in forms) |
|  |  |  |
|  |  | Help with paperwork or financial matters? |
|  |  | (Such as writing letters, sending cards, filling in forms, dealing with bills, banking) |
|  |  |  |
|  |  | Other practical help? |
|  |  | (Things like preparing meals, getting your shopping, laundry, housework, gardening, decorating, household repairs, being taken to doctor’s or hospital) |
|  |  |  |
|  |  | Having company? |
|  |  | (Things like visiting, sitting with, reading to, talking to, playing cards or games) |
|  |  |  |
|  |  | Being taken out? |
|  |  | (Such as taking out for a walk or drive, taking to see friends or relatives) |
|  |  |  |
|  |  | Help with medicines? |
|  |  | (Things like help with pills, giving injections, changing dressings) |
|  |  |  |
|  |  | Emotional support? |
|  |  |  |
|  |  |  |
|  |  | Other help? |

Thinking about the help or care you are receiving; how well are your needs being met:

| All my needs are fully met |  |
| --- | --- |
| Some of my needs are met |  |
| Very few of my needs are adequately met |  |
| None of my needs are adequately met |  |

Has the level of support you receive been affected by the COVID-19 pandemic:

| Yes – it has reduced the support I receive **slightly** |  |
| --- | --- |
| Yes – it has reduced the support I receive **a lot** |  |
| No – it has had **no impact** |  |

**** Include ASCOT self-complete SCT**

To appear at end of all questions

Please add any comments you may have:

|  |
| --- |

**Thank you for completing the survey.**

We would like to draw your attention to the following free resources, which may be useful if you require additional support:

**Samaritans** 116 123, email: [jo@samaritans.org](mailto:jo@samaritans.org)

https://[www.samaritans.org](http://www.samaritans.org)

*Charity that provides a free listening service.*

**Mind** 0300 123 3393, [info@mind.org.uk,](mailto:info@mind.org.uk,%2520) Text: 86463, https://www.mind.org.uk/information-support/

*Charity that provides advice and support to those living with mental health problems, or supporting someone who is. Offering access to the right information, about conditions, treatment options and practical issues.*

**Moodzone** (England) <https://www.nhs.uk/conditions/stress-anxiety-depression/?tabname=common-problems>

**Carers UK**, 0808 808 7777, <https://www.carersuk.org/help-and-advice/talk-to-us>

*Charity that offers expert advice, information and support to carers.*

Appendix Table 1 Measures completed at the various time points

|  | Health condition sample | General Population  sample |
| --- | --- | --- |
|  |  |  |
| EQ-HWB-25 completed @ T1 | 767 | 302 |
| EQ-HWB-25 completed @ T2 | 250 | 50 |
| SWEMWBS completed @ T1 | 767 | 302 |
| SWEMWBS completed @ T2 | 250 | 50 |
| EQ-5D-5L completed @ T1 | 767 | 302 |
| EQ-5D-5L completed @ T2 | 250 | 50 |
| ICECAP-A completed @ T1 | 767 | 302 |
| PHQ-8 completed @ T1 | 767 | 302 |
| GAD-7 completed @ T1 | 767 | 302 |
| Anxiety and Depression questions completed @ T1 | 390 | 1 |
| Diabetes questions completed @ T1 | 232 | 0 |
| Arthritis questions completed @ T1 | 234 | 0 |
| Asthma questions completed @ T1 | 187 | 0 |
| UCLA completed @T1 | 767 | 302 |
|  |  |  |

Appendix Table 2a Known-groups hypotheses

| **Known-group** | **Description** | **Hypothesis** |
| --- | --- | --- |
| **Health condition and general population samples** | |  |
| 1. Total UCLA3 Loneliness Scale score | Not lonely (3 to 5) vs lonely (6 to 9) (Surkalim 2022) | Those classified as lonely have lower health and QoL |
| 1. General health | Excellent, very good, good vs fair and poor | Those with poorer health have lower health and QoL |
| 1. Physical health impact | Accomplished less work and daily activities due to physical health  none, a little vs some, most or all of the time | Those experiencing higher impact have lower health and QoL |
| 1. Emotional health impact | Accomplished less work and daily activities due to emotional health  none, a little vs some, most or all of the time | Those experiencing higher impact have lower health and QoL |
| 1. PHQ-8 severity | No and mild (0 – 9) vs moderate and severe (≥ 10) depression | Those with higher depression symptoms have lower health and QoL. The cut point of >=10 has been found to adequately identify those with current depression in population surveys. |
| 1. GAD-7 severity | Non-clinical (0 – 9) vs clinical cut-off (≥ 10) (Kroenke 2008) | Those with higher anxiety symptoms have lower health and QoL |
| 1. Presence of long-term condition | No self-reported long-term condition  vs at least one | Those with a long-term condition have lower health and QoL |
| 1. Impact of long-term condition^a^ | None and mild v moderate and severe | Those experiencing higher impact have lower health and QoL |
| **Patient population self-reporting anxiety and/or depression** | | |
| 1. PHQ-8 severity | No and mild (0 – 9) vs moderate and severe (≥ 10) depression | Those with higher depression symptoms have lower health and QoL |
| 1. GAD-7 severity | Non-clinical (0 – 7) versus clinical cut-off (≥ 8) | Those with higher anxiety symptoms have lower health and QoL |
| **Patient population self-reporting diabetes** | | |
| 1. Number of diabetes related complications | Complications summed across the following areas: eyes, foot, kidney, nerve, gum and other | Those with higher number of complications have lower health and QoL |
| **Patient population self-reporting arthritis** | | |
| 1. Number of joints affected | Summed across the following areas: back, feet, hands/wrists, hips, knees, neck, other | Those with higher number of joints affected have lower health and QoL |
| 1. Arthritis VAS^b^ | Pain on a scale of 0 (zero) to 100 (maximum pain) in the last week | Those with higher pain levels have lower health and QoL |
| **Patient population self-reporting rheumatoid arthritis** | | |
| 1. Severity calculated across 5 questions^b^ | In the last month whether RA was active, RA active today: tenderness and swelling of joints, pain, general health and stiffness experienced yesterday. Calculated an index by using the mean of five questions | Those with active RA will have lower health and QoL |
| **Patient population self-reporting respiratory conditions (asthma, COPD and bronchitis)** | | |
| 1. At least 1 symptom | 0 symptom vs ≥ 1 symptom  (symptoms in the morning, cough and breathing problems during the day, symptoms in the night) | Those with more symptoms have lower health and QoL |
| **Self-reported social care** | | |
| 1. Unmet needs (self-reported) | All needs are fully met vs some needs, very few or no needs met | Those with more unmet needs have lower health and QoL |

Abbreviation: UCLA: University of California, Los Angeles Loneliness Scale

Note: ^a^ Participants in the patient group did not answer this question as we know they have a long-term condition from the screening question. Participants in the general population sample answered the question: “Do you have any long-standing physical or mental health impairment, illness or disability which has been diagnosed by a doctor? By 'long-standing' we mean anything that has troubled you over a period of at least 12 months or that is likely to trouble you over a period of at least 12 months”. ^b^  This is strictly speaking convergent validity and presented as thus in the paper. These are added to this table for completeness.

**References**

Kroenke K, Strine TW, Spitzer RL, Williams JB, Berry JT, Mokdad AH. The PHQ-8 as a measure of current depression in the general population. Journal of affective disorders. 2009 Apr 1;114(1-3):163-73.

Surkalim DL, Luo M, Eres R, Gebel K, Van Buskirk J, Bauman A, Ding D. The prevalence of loneliness across 113 countries: systematic review and meta-analysis. bmj. 2022 Feb 9;376.

**Appendix Table 2b Hypothesised Spearman correlations between selected EQ-HWB-25 and EQ-5D-5L items**

|  | EQ-5D-5L items | Mobility | | Self-care | | Usual activities | | Pain and discomfort | | Anxious/  depressed | |
| --- | --- | --- | --- | --- | --- | --- | --- | --- | --- | --- | --- |
| EQ-HWB | EQ-HWB description | Health condition sample | Gen pop | Health condition sample | Gen pop | Health condition sample | Gen pop | Health condition sample | Gen pop | Health condition sample | Gen pop |
| EQ-HWB3 | Getting around inside and outside | (S) | (M) |  |  |  |  |  |  |  |  |
| EQ-HWB4 | Day-to-day activities |  |  |  |  | (S) | (M) |  |  |  |  |
| EQ-HWB5 | Washing dressing eating caring appearance |  |  | (S) | (M) |  |  |  |  |  |  |
| EQ-HWB12 | Anxious |  |  |  |  |  |  |  |  | (S) | (M) |
| EQ-HWB14 | Frustrated |  |  |  |  |  |  |  |  | (M) | (M) |
| EQ-HWB15 | Sad or depressed |  |  |  |  |  |  |  |  | (S) | (M) |
| EQ-HWB16 | Nothing to look forward to |  |  |  |  |  |  |  |  | (S) | (M) |
| EQ-HWB22 | Physical pain (frequency) |  |  |  |  |  |  | (S) | (M) |  |  |
| EQ-HWB23 | Physical pain (severity) |  |  |  |  |  |  | (S) | (M) |  |  |
| EQ-HWB24 | Physical discomfort (frequency) |  |  |  |  |  |  | (S) | (M) |  |  |
| EQ-HWB25 | Physical discomfort (severity) |  |  |  |  |  |  | (S) | (M) |  |  |

**Appendix Table 2c Hypothesised Spearman correlations between selected EQ-HWB and SWEMWBS items**

Notes: (S) – Hypothesised strong correlation; (M) – hypothesised moderate correlation.

| EQ-HWB items | | SWEMWBS items | | | | | | | | | | | |
| --- | --- | --- | --- | --- | --- | --- | --- | --- | --- | --- | --- | --- | --- |
|  |  | 1 | | 3 | | 4 | | 5 | | 6 | | 7 | |
|  |  | Feeling optimistic about the future | | Feeling relaxed | | Dealing with problems well | | Thinking clearly | | Feeling close to other people | | Able to make up my own mind about things | |
| Sample | | Health condition | Gen pop | Health condition | Gen pop | Health condition | Gen pop | Health condition | Gen pop | Health condition | Gen pop | Health condition | Gen pop |
| 8 | Lonely * |  |  |  |  |  |  |  |  | (S) | (M) |  |  |
| 9 | Unsupported by people |  |  |  |  |  |  |  |  | (S) | (M) |  |  |
| 10 | Trouble remembering |  |  |  |  |  |  | (M) | (M) |  |  |  |  |
| 11 | Trouble concentrating or thinking clearly |  |  |  |  |  |  | (S) | (M) |  |  |  |  |
| 12 | Anxious* |  |  | (S) | (M) |  |  |  |  |  |  |  |  |
| 15 | Sad or depressed* | (M) | (M) |  |  |  |  |  |  |  |  |  |  |
| 16 | Nothing to look forward to | (S) | (M) |  |  |  |  |  |  |  |  |  |  |
| 17 | No control over day to day* |  |  |  |  | (M) | (M) |  |  |  |  | (M) | (M) |
| 18 | Unable to cope with my day-to-day life |  |  |  |  | (M) | (M) |  |  |  |  | (M) | (M) |
| 19 | Accepted by others |  |  |  |  |  |  |  |  | (S) | (M) |  |  |

Notes: (S) – Hypothesised strong correlation; (M) – hypothesised moderate correlation.

Appendix Table 2d Hypothesised correlations between selected EQ-HWB and ICECAP-A items

| EQ-HWB items | | ICECAP-A items | | | | | | | | | |
| --- | --- | --- | --- | --- | --- | --- | --- | --- | --- | --- | --- |
|  |  | 1 | | 2 | | 3 | | 4 | | 5 | |
|  |  | Able to feel settled and secure | | Can have love, friendship and support | | Able to be independent | | Can achieve and progress | | Can have enjoyment and pleasure | |
| Sample | | Health condition | Gen pop | Health condition | Gen pop | Health condition | Gen pop | Health condition | Gen pop | Health condition | Gen pop |
| 4 | Difficulty with day-to-day activities |  |  |  |  | (M) | (M) | (M) | (M) |  |  |
| 8 | Lonely * |  |  | (S) | (M) |  |  |  |  |  |  |
| 9 | Unsupported by people |  |  | (S) | (M) |  |  |  |  |  |  |
| 12 | Anxious* | (M) | (M) |  |  |  |  |  |  |  |  |
| 13 | Unsafe | (S) | (S) |  |  |  |  |  |  |  |  |
| 15 | Sad or depressed* |  |  |  |  |  |  |  |  | (S) | (S) |
| 16 | Nothing to look forward to |  |  |  |  |  |  |  |  | (S) | (S) |
| 17 | No control over day to day* |  |  |  |  | (M) | (M) |  |  |  |  |
| 19 | Accepted by others |  |  |  |  |  |  |  |  |  |  |
| 20 | Good about myself |  |  |  |  |  |  |  |  | (S) | (S) |
| 21 | Do the things I wanted to do |  |  |  |  | (M) | (M) | (S) | (S) | (S) | (S) |

Notes: (S) – Hypothesised strong correlation; (M) – hypothesised moderate correlation..

Appendix Table 2e Hypothesised correlations between selected EQ-HWB and ASCOT items

| EQ-HWB items | | ASCOT items | | | | | | | | | | | |
| --- | --- | --- | --- | --- | --- | --- | --- | --- | --- | --- | --- | --- | --- |
|  |  | 1 | | 2 | | 3 | | 4 | | 5 | | 6 | |
|  |  | Control over daily life | | Clean and presentable | | Food and drink | | Safety | | Social contact | | Activities | |
| Sample | | Health condition | Gen pop | Health condition | Gen pop | Health condition | Gen pop | Health condition | Gen pop | Health condition | Gen pop | Health condition | Gen pop |
| 4 | Day-to-day activities |  |  |  |  |  |  |  |  |  |  | (S) | (S) |
| 5 | Washing dressing eating caring for appearance |  |  | (M) | (M) | (M) | (M) |  |  |  |  |  |  |
| 8 | Lonely * |  |  |  |  |  |  |  |  | (S) | (S) |  |  |
| 9 | Unsupported by people |  |  |  |  |  |  |  |  | (S) | (S) |  |  |
| 12 | Anxious* |  |  |  |  |  |  | (M) | (M) |  |  |  |  |
| 13 | Unsafe |  |  |  |  |  |  | (S) | (S) |  |  |  |  |
| 17 | No control over day to day* | (S) | (S) |  |  |  |  |  |  |  |  | (M) | (M) |
| 21 | Do the things I wanted to do | (S) | (M) |  |  |  |  |  |  |  |  | (S) | (S) |

Notes: (S) – Hypothesised strong correlation; (M) – hypothesised moderate correlation.

Appendix Table 3a Frequency of EQ-HWB-25 at baseline (Health condition sample n = 767)

|  |  | Percent | | | | |
| --- | --- | --- | --- | --- | --- | --- |
|  |  | level 1 | level 2 | level 3 | level 4 | level 5 |
| EQ-HWB1 | Difficulty seeing | 62.7 | 22.3 | 12.1 | 2.5 | 0.4 |
| EQ-HWB2 | Difficulty hearing | 72.5 | 15.8 | 8.9 | 2.7 | 0.1 |
| EQ-HWB3 | Difficulty getting around inside and outside* | 59.2 | 18.8 | 13.4 | 7.4 | 1.2 |
| EQ-HWB4 | Difficulty with day-to-day activities* | 45.0 | 26.2 | 16.8 | 10.2 | 1.8 |
| EQ-HWB5 | Difficulty washing, eating, dressing, caring | 69.5 | 15.0 | 11.2 | 4.0 | 0.3 |
| EQ-HWB6 | Problems with sleep | 17.6 | 25.2 | 23.5 | 17.6 | 16.2 |
| EQ-HWB7 | Exhausted* | 17.5 | 24.5 | 25.4 | 19.2 | 13.4 |
| EQ-HWB8 | Lonely * | 43.0 | 20.1 | 19.8 | 9.4 | 7.7 |
| EQ-HWB9 | Unsupported by people | 49.3 | 21.0 | 15.0 | 8.0 | 6.8 |
| EQ-HWB10 | Trouble remembering | 40.9 | 28.8 | 17.3 | 9.1 | 3.8 |
| EQ-HWB11 | Trouble concentrating* | 39.2 | 26.7 | 18.9 | 9.5 | 5.6 |
| EQ-HWB12 | Anxious* | 30.1 | 23.7 | 21.3 | 17.1 | 7.8 |
| EQ-HWB13 | Unsafe | 66.5 | 14.2 | 11.1 | 5.6 | 2.6 |
| EQ-HWB14 | Frustrated | 30.2 | 27.1 | 23.5 | 13.3 | 5.9 |
| EQ-HWB15 | Sad or depressed* | 34.8 | 22.0 | 22.6 | 12.3 | 8.3 |
| EQ-HWB16 | Nothing to look forward to | 42.9 | 18.6 | 18.4 | 10.3 | 9.8 |
| EQ-HWB17 | No control over day to day* | 45.6 | 19.3 | 17.2 | 10.2 | 7.7 |
| EQ-HWB18 | Unable to cope with my day | 52.4 | 21.4 | 14.0 | 7.2 | 5.1 |
| EQ-HWB19 | Accepted by others | 29.1 | 26.2 | 23.5 | 14.3 | 6.9 |
| EQ-HWB20 | Good about myself | 13.2 | 20.1 | 29.2 | 25.6 | 12.0 |
| EQ-HWB21 | Do the things I wanted to do | 21.8 | 23.6 | 28.3 | 20.7 | 5.6 |
| EQ-HWB22 | Physical pain (frequency) | 21.4 | 23.9 | 22.6 | 16.8 | 15.4 |
| EQ-HWB23 | Physical pain (severity)* | 21.5 | 37.8 | 27.0 | 10.6 | 3.1 |
| EQ-HWB24 | Physical discomfort (frequency) | 31.8 | 25.6 | 20.6 | 13.6 | 8.5 |
| EQ-HWB25 | Physical discomfort (severity) | 27.0 | 38.9 | 23.5 | 8.1 | 2.6 |

Key: Level 1 – least difficulty/impairment Level 5 – most difficulty/impairment. The positively worded items have all been reverse coded, so the levels are consistent across all items.

* EQ-HWB-9 items.

Appendix Table 3b Frequency of EQ-HWB-25 at baseline (General Population sample n = 302)

|  |  | Percent | | | | |
| --- | --- | --- | --- | --- | --- | --- |
|  |  | level 1 | level 2 | level 3 | level 4 | level 5 |
| EQ-HWB1 | Seeing | 78.8 | 13.6 | 5.6 | 2.0 | 0.0 |
| EQ-HWB2 | Hearing | 79.8 | 13.9 | 4.3 | 2.0 | 0.0 |
| EQ-HWB3 | Getting around inside and outside* | 85.8 | 6.6 | 5.3 | 2.3 | 0.0 |
| EQ-HWB4 | Day to day activities* | 80.8 | 11.3 | 5.3 | 2.6 | 0.0 |
| EQ-HWB5 | Washing, eating, dressing, caring | 91.4 | 2.6 | 4.3 | 1.7 | 0.0 |
| EQ-HWB6 | Problems with sleep | 31.8 | 35.4 | 21.2 | 7.9 | 3.6 |
| EQ-HWB7 | Exhausted* | 34.8 | 31.8 | 22.5 | 8.9 | 2.0 |
| EQ-HWB8 | Lonely * | 60.9 | 19.9 | 11.9 | 5.6 | 1.7 |
| EQ-HWB9 | Unsupported by people | 65.9 | 14.6 | 12.9 | 4.6 | 2.0 |
| EQ-HWB10 | Trouble remembering | 62.9 | 22.5 | 9.3 | 4.3 | 1.0 |
| EQ-HWB11 | Trouble concentrating* | 62.3 | 21.2 | 11.9 | 4.3 | 0.3 |
| EQ-HWB12 | Anxious* | 56.0 | 24.2 | 12.9 | 5.6 | 1.3 |
| EQ-HWB13 | Unsafe | 82.8 | 9.6 | 4.3 | 2.6 | 0.7 |
| EQ-HWB14 | Frustrated | 51.7 | 29.1 | 11.3 | 6.0 | 2.0 |
| EQ-HWB15 | Sad or depressed* | 61.3 | 22.5 | 9.6 | 4.6 | 2.0 |
| EQ-HWB16 | Nothing to look forward to | 67.9 | 13.9 | 10.6 | 5.6 | 2.0 |
| EQ-HWB17 | No control over day to day* | 72.2 | 13.6 | 7.9 | 3.3 | 3.0 |
| EQ-HWB18 | Unable to cope with my day | 80.5 | 12.3 | 3.0 | 3.6 | 0.7 |
| EQ-HWB19 | Accepted by others | 31.1 | 32.1 | 17.2 | 9.9 | 9.6 |
| EQ-HWB20 | Good about myself | 23.8 | 29.8 | 22.5 | 14.6 | 9.3 |
| EQ-HWB21 | Do the things I wanted to do | 33.8 | 28.5 | 19.5 | 11.6 | 6.6 |
| EQ-HWB22 | Physical pain (frequency) | 47.0 | 28.1 | 16.2 | 5.6 | 3.0 |
| EQ-HWB23 | Physical pain (severity)* | 45.7 | 36.1 | 15.2 | 2.0 | 1.0 |
| EQ-HWB24 | Physical discomfort (frequency) | 64.6 | 22.8 | 8.9 | 3.0 | 0.7 |
| EQ-HWB25 | Physical discomfort (severity) | 56.6 | 31.8 | 9.3 | 2.3 | 0.0 |

Key: Level 1 – least difficulty/impairment Level 5 – most difficulty/impairment * EQ-HWB-9 items. The positively worded items have all been reverse coded, so the levels are consistent across all items.

**Appendix Table 4a Correlations EQHWB-25 items – health condition sample**

| Item | eqhwb1 | eqhwb2 | eqhwb3 | eqhwb4 | eqhwb5 | eqhwb6 | eqhwb7 | eqhwb8 | eqhwb9 | eqhwb10 | eqhwb11 | eqhwb12 | eqhwb13 | eqhwb14 | eqhwb15 | eqhwb16 | eqhwb17 | eqhwb18 | eqhwb19 | eqhwb20 | eqhwb21 | eqhwb22 | eqhwb23 | eqhwb24 | eqhwb25 |
| --- | --- | --- | --- | --- | --- | --- | --- | --- | --- | --- | --- | --- | --- | --- | --- | --- | --- | --- | --- | --- | --- | --- | --- | --- | --- |
| eqhwb1 | 1 |  |  |  |  |  |  |  |  |  |  |  |  |  |  |  |  |  |  |  |  |  |  |  |  |
| eqhwb2 | 0.352 | 1 |  |  |  |  |  |  |  |  |  |  |  |  |  |  |  |  |  |  |  |  |  |  |  |
| eqhwb3 | 0.384 | 0.331 | 1 |  |  |  |  |  |  |  |  |  |  |  |  |  |  |  |  |  |  |  |  |  |  |
| eqhwb4 | 0.357 | 0.223 | 0.745 | 1 |  |  |  |  |  |  |  |  |  |  |  |  |  |  |  |  |  |  |  |  |  |
| eqhwb5 | 0.347 | 0.26 | 0.69 | 0.685 | 1 |  |  |  |  |  |  |  |  |  |  |  |  |  |  |  |  |  |  |  |  |
| eqhwb6 | 0.256 | 0.108 | 0.277 | 0.382 | 0.318 | 1 |  |  |  |  |  |  |  |  |  |  |  |  |  |  |  |  |  |  |  |
| eqhwb7 | 0.266 | 0.139 | 0.403 | 0.494 | 0.426 | 0.659 | 1 |  |  |  |  |  |  |  |  |  |  |  |  |  |  |  |  |  |  |
| eqhwb8 | 0.278 | 0.081 | 0.266 | 0.34 | 0.317 | 0.395 | 0.493 | 1 |  |  |  |  |  |  |  |  |  |  |  |  |  |  |  |  |  |
| eqhwb9 | 0.279 | 0.122 | 0.284 | 0.341 | 0.347 | 0.404 | 0.468 | 0.717 | 1 |  |  |  |  |  |  |  |  |  |  |  |  |  |  |  |  |
| eqhwb10 | 0.363 | 0.248 | 0.335 | 0.375 | 0.354 | 0.479 | 0.539 | 0.422 | 0.46 | 1 |  |  |  |  |  |  |  |  |  |  |  |  |  |  |  |
| eqhwb11 | 0.317 | 0.18 | 0.337 | 0.433 | 0.374 | 0.548 | 0.648 | 0.53 | 0.553 | 0.768 | 1 |  |  |  |  |  |  |  |  |  |  |  |  |  |  |
| eqhwb12 | 0.246 | 0.091 | 0.242 | 0.365 | 0.33 | 0.486 | 0.592 | 0.593 | 0.539 | 0.509 | 0.655 | 1 |  |  |  |  |  |  |  |  |  |  |  |  |  |
| eqhwb13 | 0.344 | 0.196 | 0.434 | 0.454 | 0.505 | 0.371 | 0.475 | 0.511 | 0.49 | 0.474 | 0.509 | 0.57 | 1 |  |  |  |  |  |  |  |  |  |  |  |  |
| eqhwb14 | 0.301 | 0.108 | 0.391 | 0.49 | 0.406 | 0.501 | 0.623 | 0.567 | 0.551 | 0.5 | 0.613 | 0.66 | 0.564 | 1 |  |  |  |  |  |  |  |  |  |  |  |
| eqhwb15 | 0.265 | 0.104 | 0.313 | 0.429 | 0.368 | 0.506 | 0.625 | 0.692 | 0.62 | 0.487 | 0.633 | 0.767 | 0.55 | 0.726 | 1 |  |  |  |  |  |  |  |  |  |  |
| eqhwb16 | 0.272 | 0.102 | 0.324 | 0.416 | 0.375 | 0.459 | 0.571 | 0.671 | 0.649 | 0.491 | 0.592 | 0.675 | 0.546 | 0.658 | 0.803 | 1 |  |  |  |  |  |  |  |  |  |
| eqhwb17 | 0.317 | 0.129 | 0.395 | 0.499 | 0.438 | 0.496 | 0.607 | 0.608 | 0.606 | 0.478 | 0.591 | 0.667 | 0.601 | 0.705 | 0.729 | 0.76 | 1 |  |  |  |  |  |  |  |  |
| eqhwb18 | 0.33 | 0.165 | 0.408 | 0.523 | 0.49 | 0.474 | 0.591 | 0.598 | 0.535 | 0.513 | 0.645 | 0.707 | 0.631 | 0.654 | 0.725 | 0.714 | 0.769 | 1 |  |  |  |  |  |  |  |
| eqhwb19 | 0.196 | 0.064 | 0.217 | 0.295 | 0.295 | 0.294 | 0.362 | 0.548 | 0.532 | 0.317 | 0.389 | 0.486 | 0.424 | 0.429 | 0.533 | 0.54 | 0.499 | 0.459 | 1 |  |  |  |  |  |  |
| eqhwb20 | 0.182 | 0.039 | 0.27 | 0.397 | 0.299 | 0.452 | 0.533 | 0.523 | 0.477 | 0.377 | 0.475 | 0.574 | 0.386 | 0.536 | 0.653 | 0.643 | 0.576 | 0.527 | 0.641 | 1 |  |  |  |  |  |
| eqhwb21 | 0.267 | 0.115 | 0.439 | 0.508 | 0.426 | 0.426 | 0.535 | 0.461 | 0.431 | 0.419 | 0.476 | 0.477 | 0.422 | 0.519 | 0.54 | 0.54 | 0.588 | 0.526 | 0.558 | 0.688 | 1 |  |  |  |  |
| eqhwb22 | 0.276 | 0.185 | 0.579 | 0.533 | 0.435 | 0.396 | 0.447 | 0.205 | 0.229 | 0.322 | 0.334 | 0.231 | 0.363 | 0.39 | 0.29 | 0.293 | 0.307 | 0.335 | 0.129 | 0.29 | 0.351 | 1 |  |  |  |
| eqhwb23 | 0.257 | 0.177 | 0.597 | 0.551 | 0.46 | 0.369 | 0.427 | 0.186 | 0.213 | 0.314 | 0.323 | 0.202 | 0.36 | 0.371 | 0.271 | 0.257 | 0.313 | 0.327 | 0.114 | 0.251 | 0.349 | 0.843 | 1 |  |  |
| eqhwb24 | 0.278 | 0.161 | 0.526 | 0.538 | 0.441 | 0.391 | 0.499 | 0.311 | 0.292 | 0.336 | 0.4 | 0.319 | 0.387 | 0.453 | 0.355 | 0.391 | 0.41 | 0.396 | 0.234 | 0.37 | 0.411 | 0.66 | 0.626 | 1 |  |
| eqhwb25 | 0.287 | 0.191 | 0.568 | 0.551 | 0.487 | 0.353 | 0.447 | 0.252 | 0.265 | 0.313 | 0.355 | 0.258 | 0.403 | 0.394 | 0.311 | 0.34 | 0.371 | 0.374 | 0.193 | 0.298 | 0.373 | 0.689 | 0.737 | 0.793 | 1 |

| Items | eqhwb1 | eqhwb2 | eqhwb3 | eqhwb4 | eqhwb5 | eqhwb6 | eqhwb7 | eqhwb8 | eqhwb9 | eqhwb10 | eqhwb11 | eqhwb12 | eqhwb13 | eqhwb14 | eqhwb15 | eqhwb16 | eqhwb17 | eqhwb18 | eqhwb19 | eqhwb20 | eqhwb21 | eqhwb22 | eqhwb23 | eqhwb24 | eqhwb25 |
| --- | --- | --- | --- | --- | --- | --- | --- | --- | --- | --- | --- | --- | --- | --- | --- | --- | --- | --- | --- | --- | --- | --- | --- | --- | --- |
| eqhwb1 | 1 |  |  |  |  |  |  |  |  |  |  |  |  |  |  |  |  |  |  |  |  |  |  |  |  |
| eqhwb2 | 0.465 | 1 |  |  |  |  |  |  |  |  |  |  |  |  |  |  |  |  |  |  |  |  |  |  |  |
| eqhwb3 | 0.293 | 0.334 | 1 |  |  |  |  |  |  |  |  |  |  |  |  |  |  |  |  |  |  |  |  |  |  |
| eqhwb4 | 0.346 | 0.348 | 0.729 | 1 |  |  |  |  |  |  |  |  |  |  |  |  |  |  |  |  |  |  |  |  |  |
| eqhwb5 | 0.284 | 0.327 | 0.743 | 0.614 | 1 |  |  |  |  |  |  |  |  |  |  |  |  |  |  |  |  |  |  |  |  |
| eqhwb6 | 0.225 | 0.213 | 0.242 | 0.32 | 0.213 | 1 |  |  |  |  |  |  |  |  |  |  |  |  |  |  |  |  |  |  |  |
| eqhwb7 | 0.219 | 0.185 | 0.258 | 0.35 | 0.249 | 0.673 | 1 |  |  |  |  |  |  |  |  |  |  |  |  |  |  |  |  |  |  |
| eqhwb8 | 0.276 | 0.197 | 0.238 | 0.278 | 0.224 | 0.41 | 0.452 | 1 |  |  |  |  |  |  |  |  |  |  |  |  |  |  |  |  |  |
| eqhwb9 | 0.25 | 0.2 | 0.176 | 0.25 | 0.229 | 0.347 | 0.434 | 0.667 | 1 |  |  |  |  |  |  |  |  |  |  |  |  |  |  |  |  |
| eqhwb10 | 0.331 | 0.264 | 0.287 | 0.231 | 0.246 | 0.376 | 0.417 | 0.395 | 0.349 | 1 |  |  |  |  |  |  |  |  |  |  |  |  |  |  |  |
| eqhwb11 | 0.38 | 0.231 | 0.27 | 0.268 | 0.248 | 0.467 | 0.554 | 0.502 | 0.467 | 0.702 | 1 |  |  |  |  |  |  |  |  |  |  |  |  |  |  |
| eqhwb12 | 0.232 | 0.14 | 0.162 | 0.22 | 0.171 | 0.421 | 0.545 | 0.508 | 0.555 | 0.399 | 0.585 | 1 |  |  |  |  |  |  |  |  |  |  |  |  |  |
| eqhwb13 | 0.318 | 0.186 | 0.32 | 0.343 | 0.379 | 0.356 | 0.409 | 0.444 | 0.463 | 0.382 | 0.491 | 0.513 | 1 |  |  |  |  |  |  |  |  |  |  |  |  |
| eqhwb14 | 0.252 | 0.157 | 0.224 | 0.283 | 0.217 | 0.447 | 0.541 | 0.56 | 0.564 | 0.378 | 0.519 | 0.68 | 0.51 | 1 |  |  |  |  |  |  |  |  |  |  |  |
| eqhwb15 | 0.271 | 0.234 | 0.201 | 0.291 | 0.203 | 0.468 | 0.559 | 0.649 | 0.611 | 0.464 | 0.62 | 0.731 | 0.5 | 0.696 | 1 |  |  |  |  |  |  |  |  |  |  |
| eqhwb16 | 0.22 | 0.177 | 0.234 | 0.279 | 0.245 | 0.384 | 0.486 | 0.634 | 0.639 | 0.434 | 0.558 | 0.648 | 0.504 | 0.627 | 0.699 | 1 |  |  |  |  |  |  |  |  |  |
| eqhwb17 | 0.298 | 0.273 | 0.328 | 0.368 | 0.344 | 0.413 | 0.505 | 0.552 | 0.542 | 0.36 | 0.553 | 0.56 | 0.545 | 0.637 | 0.642 | 0.699 | 1 |  |  |  |  |  |  |  |  |
| eqhwb18 | 0.302 | 0.194 | 0.37 | 0.427 | 0.385 | 0.464 | 0.486 | 0.535 | 0.53 | 0.449 | 0.612 | 0.563 | 0.572 | 0.581 | 0.626 | 0.613 | 0.688 | 1 |  |  |  |  |  |  |  |
| eqhwb19 | 0.136 | 0.099 | -0.013 | 0.037 | 0.069 | 0.145 | 0.186 | 0.376 | 0.382 | 0.16 | 0.307 | 0.284 | 0.21 | 0.249 | 0.365 | 0.418 | 0.31 | 0.249 | 1 |  |  |  |  |  |  |
| eqhwb20 | 0.126 | 0.127 | 0.153 | 0.177 | 0.178 | 0.269 | 0.345 | 0.371 | 0.38 | 0.242 | 0.43 | 0.392 | 0.226 | 0.347 | 0.442 | 0.477 | 0.363 | 0.379 | 0.675 | 1 |  |  |  |  |  |
| eqhwb21 | 0.186 | 0.133 | 0.239 | 0.243 | 0.203 | 0.216 | 0.319 | 0.355 | 0.361 | 0.261 | 0.394 | 0.355 | 0.255 | 0.34 | 0.368 | 0.395 | 0.367 | 0.343 | 0.63 | 0.733 | 1 |  |  |  |  |
| eqhwb22 | 0.196 | 0.304 | 0.427 | 0.505 | 0.302 | 0.33 | 0.376 | 0.253 | 0.172 | 0.301 | 0.308 | 0.305 | 0.255 | 0.297 | 0.354 | 0.255 | 0.296 | 0.345 | -0.036 | 0.098 | 0.081 | 1 |  |  |  |
| eqhwb23 | 0.21 | 0.317 | 0.392 | 0.429 | 0.317 | 0.281 | 0.326 | 0.195 | 0.179 | 0.283 | 0.263 | 0.252 | 0.23 | 0.274 | 0.297 | 0.221 | 0.276 | 0.315 | 0.034 | 0.159 | 0.157 | 0.833 | 1 |  |  |
| eqhwb24 | 0.266 | 0.216 | 0.358 | 0.373 | 0.309 | 0.295 | 0.348 | 0.341 | 0.282 | 0.266 | 0.394 | 0.375 | 0.348 | 0.401 | 0.36 | 0.389 | 0.412 | 0.464 | 0.151 | 0.195 | 0.208 | 0.525 | 0.536 | 1 |  |
| eqhwb25 | 0.225 | 0.238 | 0.411 | 0.403 | 0.352 | 0.234 | 0.326 | 0.275 | 0.269 | 0.276 | 0.338 | 0.268 | 0.253 | 0.286 | 0.295 | 0.303 | 0.374 | 0.399 | 0.107 | 0.225 | 0.219 | 0.601 | 0.677 | 0.731 | 1 |

**Appendix Table 4b Correlations EQHWB-25 items – general population sample**

=

**Appendix Table 4c Hypothesised Spearman correlations between selected EQ-HWB-25 and EQ-5D-5L items**

|  | EQ-5D-5L items | Mobility | | Self-care | | Usual activities | | Pain and discomfort | | Anxious/  depressed | |
| --- | --- | --- | --- | --- | --- | --- | --- | --- | --- | --- | --- |
| EQ-HWB | EQ-HWB description | Health condition sample | Gen pop | Health condition sample | Gen pop | Health condition sample | Gen pop | Health condition sample | Gen pop | Health condition sample | Gen pop |
| EQ-HWB3 | Getting around inside and outside | 0.776  (S) | 0.633 (M) |  |  |  |  |  |  |  |  |
| EQ-HWB4 | Day-to-day activities |  |  |  |  | 0.808  (S) | 0.646  (M) |  |  |  |  |
| EQ-HWB5 | Washing dressing eating caring appearance |  |  | 0.828  (S) | 0.653  (M) |  |  |  |  |  |  |
| EQ-HWB12 | Anxious |  |  |  |  |  |  |  |  | 0.762  (S) | 0.668  (M) |
| EQ-HWB14 | Frustrated |  |  |  |  |  |  |  |  | 0.625  (M) | 0.546  (M) |
| EQ-HWB15 | Sad or depressed |  |  |  |  |  |  |  |  | 0.786  (S) | 0.647  (M) |
| EQ-HWB16 | Nothing to look forward to |  |  |  |  |  |  |  |  | 0.706  (S) | 0.613  (M) |
| EQ-HWB22 | Physical pain (frequency) |  |  |  |  |  |  | 0.809  (S) | 0.691  (M) |  |  |
| EQ-HWB23 | Physical pain (severity) |  |  |  |  |  |  | 0.845  (S) | 0.710  (M) |  |  |
| EQ-HWB24 | Physical discomfort (frequency) |  |  |  |  |  |  | 0.615  (S) | 0.399  (M) |  |  |
| EQ-HWB25 | Physical discomfort (severity) |  |  |  |  |  |  | 0.684  (S) | 0.550  (M) |  |  |

**Appendix Table 4d Hypothesised Spearman correlations between selected EQ-HWB and SWEMWBS items**

Notes: Sample size - General population n = 767 Health condition population n = 302; As per the Methods section, correlations are assessed as follows: <= 0.4 – weak correlation (in red); > 0.4 – < 0.7 as moderate correlation; ≥ 0·7 – strong correlation (in green). (S) – Hypothesised strong correlation; (M) – hypothesised moderate correlation. Highlighted in yellow: instances where the expected strength of correlation was observed.

| EQ-HWB items | | SWEMWBS items | | | | | | | | | | | |
| --- | --- | --- | --- | --- | --- | --- | --- | --- | --- | --- | --- | --- | --- |
|  |  | 1 | | 3 | | 4 | | 5 | | 6 | | 7 | |
|  |  | Feeling optimistic about the future | | Feeling relaxed | | Dealing with problems well | | Thinking clearly | | Feeling close to other people | | Able to make up my own mind about things | |
| Sample | | Health condition | Gen pop | Health condition | Gen pop | Health condition | Gen pop | Health condition | Gen pop | Health condition | Gen pop | Health condition | Gen pop |
| 8 | Lonely * |  |  |  |  |  |  |  |  | -0.562  (S) | -0.406  (M) |  |  |
| 9 | Unsupported by people |  |  |  |  |  |  |  |  | -0.540  (S) | -0.409  (M) |  |  |
| 10 | Trouble remembering |  |  |  |  |  |  | -0.517  (M) | -0.417  (M) |  |  |  |  |
| 11 | Trouble concentrating or thinking clearly |  |  |  |  |  |  | -0.651  (S) | -0.572  (M) |  |  |  |  |
| 12 | Anxious* |  |  | -0.651  (S) | -0.523  (M) |  |  |  |  |  |  |  |  |
| 15 | Sad or depressed* | -0.566  (M) | -0.418  (M) |  |  |  |  |  |  |  |  |  |  |
| 16 | Nothing to look forward to | -0.586  (S) | -0.413  (M) |  |  |  |  |  |  |  |  |  |  |
| 17 | No control over day to day* |  |  |  |  | -0.535  (M) | -0.385  (M) |  |  |  |  | -0.484  (M) | -0.259  (M) |
| 18 | Unable to cope with my day-to-day life |  |  |  |  | -0.528  (M) | -0.393  (M) |  |  |  |  | -0.507  (M) | -0.377  (M) |
| 19 | Accepted by others |  |  |  |  |  |  |  |  | -0.635  (S) | -0.451  (M) |  |  |

Notes: Sample size - General population n = 767 Patients population n = 302; As per the Methods section, correlations are assessed as follows: <= 0.4 – weak correlation (in red); > 0.4 – < 0.7 as moderate correlation; ≥ 0·7 – strong correlation (in green). (S) – Hypothesised strong correlation; (M) – hypothesised moderate correlation. Highlighted in yellow: instances where the expected strength of correlation was observed.

Appendix Table 4e Hypothesised correlations between selected EQ-HWB and ICECAP-A items

| EQ-HWB items | | ICECAP-A items | | | | | | | | | |
| --- | --- | --- | --- | --- | --- | --- | --- | --- | --- | --- | --- |
|  |  | 1 | | 2 | | 3 | | 4 | | 5 | |
|  |  | Able to feel settled and secure | | Can have love, friendship and support | | Able to be independent | | Can achieve and progress | | Can have enjoyment and pleasure | |
| Sample | | Health condition | Gen pop | Health condition | Gen pop | Health condition | Gen pop | Health condition | Gen pop | Health condition | Gen pop |
| 4 | Difficulty with day-to-day activities |  |  |  |  | -0.541  (M) | -0.314  (M) | -0.466  (M) | -0.258  (M) |  |  |
| 8 | Lonely * |  |  | -0.576  (S) | -0.492  (M) |  |  |  |  |  |  |
| 9 | Unsupported by people |  |  | -0.560  (S) | -0.443  (M) |  |  |  |  |  |  |
| 12 | Anxious* | -0.581  (M) | -0.514  (M) |  |  |  |  |  |  |  |  |
| 13 | Unsafe | -0.406  (S) | -0.316  (S) |  |  |  |  |  |  |  |  |
| 15 | Sad or depressed* |  |  |  |  |  |  |  |  | -0.646  (S) | -0.501  (S) |
| 16 | Nothing to look forward to |  |  |  |  |  |  |  |  | -0.673  (S) | -0.511  (S) |
| 17 | No control over day to day* |  |  |  |  | -0.440  (M) | -0.316  (M) |  |  |  |  |
| 19 | Accepted by others |  |  |  |  |  |  |  |  |  |  |
| 20 | Good about myself |  |  |  |  |  |  |  |  | -0.684  (S) | -0.416  (S) |
| 21 | Do the things I wanted to do |  |  |  |  | -0.625  (M) | -0.295  (M) | -0.669  (S) | -0.441  (S) | -0.595  (S) | -0.358  (S) |

Notes: Sample size – Health sample population n = 767 General population = 302; As per the Methods section, correlations are assessed as follows : <= 0.4 – weak correlation (in red); > 0.4 – < 0.7 as moderate correlation ; ≥ 0·7 – strong correlation (in green). (S) – Hypothesised strong correlation; (M) – hypothesised moderate correlation. Highlighted in yellow: instances where the expected strength of correlation was observed.

Appendix Table 4f Hypothesised correlations between selected EQ-HWB and ASCOT items

| EQ-HWB items | | ASCOT items | | | | | | | | | | | |
| --- | --- | --- | --- | --- | --- | --- | --- | --- | --- | --- | --- | --- | --- |
|  |  | 1 | | 2 | | 3 | | 4 | | 5 | | 6 | |
|  |  | Control over daily life | | Clean and presentable | | Food and drink | | Safety | | Social contact | | Activities | |
| Sample | | Health condition | Gen pop | Health condition | Gen pop | Health condition | Gen pop | Health condition | Gen pop | Health condition | Gen pop | Health condition | Gen pop |
| 4 | Day-to-day activities |  |  |  |  |  |  |  |  |  |  | 0.385  (S) | 0.272  (S) |
| 5 | Washing dressing eating caring for appearance |  |  | 0.447  (M) | 0.353  (M) | 0.298  (M) | 0.599  (M) |  |  |  |  |  |  |
| 8 | Lonely * |  |  |  |  |  |  |  |  | 0.462  (S) | 0.395  (S) |  |  |
| 9 | Unsupported by people |  |  |  |  |  |  |  |  | 0.352  (S) | 0.490  (S) |  |  |
| 12 | Anxious* |  |  |  |  |  |  | 0.435  (M) | 0.689  (M) |  |  |  |  |
| 13 | Unsafe |  |  |  |  |  |  | 0.493  (S) | 0.719  (S) |  |  |  |  |
| 17 | No control over day to day* | 0.535  (S) | 0.611  (S) |  |  |  |  |  |  |  |  | 0.605  (M) | 0.579  (M) |
| 21 | Do the things I wanted to do | 0.523  (S) | 0.323  (M) |  |  |  |  |  |  |  |  | 0.562  (S) | 0.091  (S) |

Notes: Sample size – Health sample population n = 197 General population = 22; As per the Methods section, correlations are assessed as follows: <= 0.4 – weak correlation (in red); > 0.4 – < 0.7 as moderate correlation; ≥ 0·7 – strong correlation (in green). (S) – Hypothesised strong correlation; (M) – hypothesised moderate correlation. Highlighted in yellow: instances where the expected strength of correlation was observed.

Appendix Table 5a Known-group validity for the EQ-HWB measures (health condition sample)

|  |  | **Standardised Effect Size (Glass d)** | | | | | | | |
| --- | --- | --- | --- | --- | --- | --- | --- | --- | --- |
|  | **n** | **EQ-HWB-25^a^** | **EQ-HWB-9^a^** | **EQ-HWB-S** | **SWEMWBS** | **EQ-5D-5L** | **ICECAP-A** | **PHQ-8** | **GAD-7** |
|  |  | **Summative score** | | **Utility score** |  |  |  |  |  |
| **Using UCLA scores** | | | | | | | | | |
| Not lonely (score <6) | 429 | -1.36 | -1.39 | 1.22 | 1.21 | 0.66 | 1.48 | -1.36 | -1.17 |
| Lonely (score ≥ 6) | 338 |  |  |  |  |  |  |  |  |
| **Using general health** | | | | | | | | | |
| Excellent, Very good and good | 384 | -1.16 | -1.06 | 1.25 | 0.78 | 1.25 | 1.10 | -0.73 | -0.55 |
| Fair and Poor | 383 |  |  |  |  |  |  |  |  |
| **Accomplished less work and daily activities due to health problem** | | | | | | | | | |
| None and a little (of the time) | 413 | -1.02 | -0.96 | 1.06 | 0.53 | 0.98 | 0.77 | -0.82 | -0.55 |
| Some, most and all (of the time) | 354 |  |  |  |  |  |  |  |  |
| **Accomplished less work and daily activities due to emotional problems** | | | | | | | | | |
| None and a little (of the time) | 479 | -1.42 | -1.49 | 1.48 | 1.16 | 1.07 | 1.35 | -1.52 | -1.50 |
| Some, most and all (of the time) | 288 |  |  |  |  |  |  |  |  |
| **Using cut-offs from PHQ-8** | | | | | | | | | |
| Non clinical (score 0 -9) | 512 | -2.29 | -2.32 | 2.19 | 1.46 | 1.51 | 1.75 |  | -2.70 |
| Clinical (score ≥ 10) | 255 |  |  |  |  |  |  |  |  |
| **Using cut-offs from GAD-7** | | | | | | | | | |
| Non clinical (score 0 -9) | 562 | -1.99 | -2.00 | 1.90 | 1.38 | 1.37 | 1.56 | -2.42 |  |
| Clinical (score ≥ 10) | 205 |  |  |  |  |  |  |  |  |

**^a^  Sum scores are not recommended but were used for analysis purposes; Glass d cut-off used** 0.2-0.49 considered small (in red), 0.5-0.79 moderate (amber), >= 0.8 large

Appendix Table 5b Known-group validity for the EQ-HWB measures (general population)

|  |  | **Standardised Effect Size (Glass d)** | | | | | | | |
| --- | --- | --- | --- | --- | --- | --- | --- | --- | --- |
|  | **n** | **EQ-HWB** | **EQ-HWB-S** | **EQ-HWB-S** | **SWEMWBS** | **EQ-5D-5L** | **ICECAP-A** | **PHQ-8** | **GAD-7** |
|  |  | **Summative scores** | | **Utility score** |  |  |  |  |  |
| **Using UCLA scores** | | | | | | | | | |
| Not lonely (score <6) | 213 | -1.72 | -1.73 | 1.36 | 0.96 | 0.78 | 1.54 | -1.39 | -1.48 |
| Lonely (score ≥ 6) | 89 |  |  |  |  |  |  |  |  |
| **Using general health** | | | | | | | | | |
| Excellent, Very good and good | 257 | -1.31 | -1.31 | 1.63 | 0.49 | 1.81 | 0.99 | -0.87 | -0.67 |
| Fair and Poor | 45 |  |  |  |  |  |  |  |  |
| **Accomplished less work and daily activities due to health problem** | | | | | | | | | |
| None and a little (of the time) | 219 | -1.25 | -1.23 | 1.28 | 0.67 | 1.16 | 0.79 | -1.10 | -1.16 |
| Some, most and all (of the time) | 83 |  |  |  |  |  |  |  |  |
| **Accomplished less work and daily activities due to emotional problems** | | | | | | | | |  |
| None and a little (of the time) | 242 | -1.79 | -1.75 | 1.51 | 1.00 | 0.68 | 1.59 | -1.54 | -1.41 |
| Some, most and all (of the time) | 60 |  |  |  |  |  |  |  |  |
| **Presence of at least one long-term condition** | | | | | | | | | |
| No | 254 | -0.82 | -0.89 | 1.13 | 0.26 | 1.44 | 0.36 | -0.58 | -0.25 |
| Yes | 48 |  |  |  |  |  |  |  |  |
| **Using cut-offs from PHQ-8** | | | | | | | | | |
| Non clinical (score 0 -9) | 269 | -2.73 | -2.88 | 3.57 | 1.41 | 1.72 | 2.03 |  | -2.99 |
| Clinical (score ≥ 10) | 33 |  |  |  |  |  |  |  |  |
| **Using cut-offs from GAD-7** | | | | | | | | | |
| Non clinical (score 0 -9) | 282 | -2.38 | -2.43 | 2.33 | 1.50 | 1.46 | 1.98 | -2.79 |  |
| Clinical (score ≥ 10) | 20 |  |  |  |  |  |  |  |  |

**Cut-off used:** 0.2-0.49 considered small (in red), 0.5-0.79 moderate (amber), >= 0.8 large

| Appendix Table 6 Known-group validity for the EQ-HWB measures for the health condition and general population combined | | | | | | | | | | | | | | | | | | | | | | | | | | | | | | | | | | | | | | | | | | | |  |  |
| --- | --- | --- | --- | --- | --- | --- | --- | --- | --- | --- | --- | --- | --- | --- | --- | --- | --- | --- | --- | --- | --- | --- | --- | --- | --- | --- | --- | --- | --- | --- | --- | --- | --- | --- | --- | --- | --- | --- | --- | --- | --- | --- | --- | --- | --- |
|  | | | | | | | | | | | | | | | | | | | | | | | | | | | | | | | | | | | | | | | | | | | |  |  |
|  |  | **EQ-HWB-25^a^** | | | **EQ-HWB-9^a^** | | | | **EQ-HWB-S^b^** | | | | | | **SWEMWBS** | | | | | | **EQ-5D-5L** | | | | | | **ICECAP-A** | | | | | | **PHQ-8** | | | | | **GAD-7** | | | | | | | |
|  | N | Mean | SD | SES | Mean | SD | SES | | Mean | | SD | | SES | | Mean | | SD | | SES | | Mean | | SD | | SES | | Mean | | SD | | SES | | Mean | | SD | | SES | Mean | | | SD | | SES | | |
| **Using UCLA scores** |  |  |  |  |  |  |  | |  | |  | |  | |  | |  | |  | |  | |  | |  | |  | |  | |  | |  | |  | |  |  | | |  | |  | | |
| Not lonely (score <6) | 642 | 43.05 | 14.57 | -1.47 | 15.15 | 5.89 | -1.49 | | 0.83 | | 0.19 | | 1.30 | | 26.16 | | 5.14 | | 1.18 | | 0.77 | | 0.21 | | 0.75 | | 0.85 | | 0.16 | | 1.55 | | 3.61 | | 4.68 | | -1.42 | 2.73 | | | 4.31 | | -1.27 | | |
| Lonely (score ≥ 6) | 427 | 64.48 | 19.23 |  | 23.94 | 7.80 |  | | 0.58 | | 0.29 | |  | | 20.11 | | 5.38 | |  | | 0.61 | | 0.28 | |  | | 0.61 | | 0.24 | |  | | 10.26 | | 6.59 | |  | 8.22 | | | 6.22 | |  | | |
| **Using general health** |  |  |  |  |  |  |  | |  | |  | |  | |  | |  | |  | |  | |  | |  | |  | |  | |  | |  | |  | |  |  | | |  | |  | | |
| Excellent, Very good and good | 641 | 43.71 | 15.09 | -1.31 | 15.52 | 6.17 | -1.27 | | 0.84 | | 0.18 | | 1.50 | | 25.51 | | 5.59 | | 0.79 | | 0.82 | | 0.17 | | 1.54 | | 0.84 | | 0.18 | | 1.17 | | 4.30 | | 5.35 | | -0.92 | 3.49 | | | 4.90 | | -0.73 | | |
| Fair and Poor | 428 | 63.44 | 19.70 |  | 23.37 | 8.07 |  | | 0.57 | | 0.29 | |  | | 21.11 | | 5.66 | |  | | 0.55 | | 0.28 | |  | | 0.63 | | 0.24 | |  | | 9.21 | | 6.74 | |  | 7.06 | | | 6.40 | |  | | |
| **Accomplished less work and daily activities due to physical health problem** | | | | | | | | | | | | | | | | | | | | | | | | | | | | | | | | | | | | | | | | | | | |  |  |
| None and a little (of the time) | 632 | 44.17 | 15.96 | -1.14 | 15.73 | 6.56 | -1.09 | | 0.83 | | 0.20 | | 1.12 | | 25.17 | | 5.71 | | 0.61 | | 0.80 | | 0.20 | | 1.09 | | 0.82 | | 0.19 | | 0.84 | | 4.25 | | 5.22 | | -0.95 | 3.47 | | | 4.95 | | -0.72 | | |
| Some, most and all (of the time) | 437 | 62.37 | 19.46 |  | 22.90 | 7.95 |  | | 0.59 | | 0.28 | |  | | 21.69 | | 5.85 | |  | | 0.58 | | 0.28 | |  | | 0.66 | | 0.24 | |  | | 9.19 | | 6.84 | |  | 7.02 | | | 6.32 | |  | | |
| **Accomplished less work and daily activities due to emotional problems** | | | | | | | | | | | | | | | | | | | | | | | | | | | | | | | | | | | | | | | | | | | |  |  |
| None and a little (of the time) | 721 | 44.04 | 14.81 | -1.57 | 15.51 | 5.95 | -1.62 | | 0.83 | | 0.19 | | 1.58 | | 25.70 | | 5.17 | | 1.16 | | 0.78 | | 0.20 | | 1.12 | | 0.83 | | 0.17 | | 1.47 | | 3.82 | | 4.63 | | -1.63 | 2.75 | | | 4.20 | | -1.59 | | |
| Some, most and all (of the time) | 348 | 67.30 | 19.10 |  | 25.19 | 7.98 |  | | 0.53 | | 0.29 | |  | | 19.70 | | 5.62 | |  | | 0.56 | | 0.30 | |  | | 0.59 | | 0.25 | |  | | 11.34 | | 6.62 | |  | 9.42 | | | 6.12 | |  | | |
| **Presence of at least one long-term condition** | | | | | | | | | | | | | | | | | | | | | | | | | | | | | | | | | | | | | | | | | | | |  |  |
| No | 254 | 40.09 | 13.40 | -1.13 | 13.80 | 5.33 | -1.20 | | 0.89 | | 0.15 | | 1.40 | | 25.76 | | 5.56 | | 0.48 | | 0.89 | | 0.13 | | 1.81 | | 0.84 | | 0.18 | | 0.66 | | 2.93 | | 4.41 | | -0.99 | 2.28 | | | 3.97 | | -0.87 | | |
| Yes | 815 | 55.20 | 19.88 |  | 20.18 | 8.06 |  | | 0.68 | | 0.27 | |  | | 23.12 | | 6.02 | |  | | 0.65 | | 0.26 | |  | | 0.72 | | 0.24 | |  | | 7.31 | | 6.58 | |  | 5.74 | | | 6.05 | |  | | |
| **Using cut-offs from PHQ-8** | | | | | | | | | | | | | | | | | | | | | | | | | | | | | | | | | | | | | | | | | | | | |  |
| Non-clinical (score 0 -9) | 781 | 43.07 | 12.82 | -2.47 | 15.16 | 5.15 | -2.52 | | 0.84 | | 0.16 | | 2.51 | | 25.70 | | 4.90 | | 1.48 | | 0.79 | | 0.18 | | 1.69 | | 0.83 | | 0.16 | | 1.88 | |  | |  | |  | 2.34 | | | 3.30 | | -2.90 | | |
| Clinical (score ≥ 10) | 288 | 74.79 | 15.78 |  | 28.16 | 6.40 |  | | 0.44 | | 0.27 | |  | | 18.45 | | 5.55 | |  | | 0.48 | | 0.29 | |  | | 0.53 | | 0.23 | |  | |  | |  | |  | 11.90 | | | 5.43 | |  | | |
| **Using cut-offs from GAD-7** | | | | | | | | | | | | | | | | | | | | | | | | | | | | | | | | | | | | | | | | | | | | |  |
| Non-clinical (score 0 -9) | 844 | 45.05 | 14.45 | -2.16 | 15.97 | 5.87 | -2.18 | | 0.81 | | 0.17 | | 2.14 | | 25.27 | | 5.10 | | 1.42 | | 0.77 | | 0.20 | | 1.56 | | 0.82 | | 0.18 | | 1.70 | | 3.94 | | 4.25 | | -2.58 |  | | |  | |  | | |
| Clinical (score ≥ 10) | 225 | 76.23 | 16.74 |  | 28.76 | 6.62 |  | | 0.42 | | 0.28 | |  | | 18.02 | | 5.76 | |  | | 0.46 | | 0.30 | |  | | 0.52 | | 0.24 | |  | | 15.00 | | 5.47 | |  |  | | |  | |  | | |
| **Impact of conditions/symptoms** | | | | | | | |  | |  | |  | |  | |  | |  | |  | |  | |  | |  | |  | |  | |  | |  | |  | | |  |  | |  | |  |  |
| None and mild | 411 | 45.21 | 14.57 | -1.40 | 16.15 | 5.89 | -1.39 | | 0.82 | | 0.17 | | 1.64 | | 25.15 | | 5.33 | | 0.80 | | 0.79 | | 0.17 | | 1.63 | | 0.82 | | 0.18 | | 1.11 | | 4.38 | | 4.89 | | -1.23 | 3.35 | | | 4.27 | | -1.15 | | |
| Moderate and severe | 388 | 65.58 | 19.24 |  | 24.35 | 7.86 |  | | 0.54 | | 0.28 | |  | | 20.88 | | 5.92 | |  | | 0.51 | | 0.27 | |  | | 0.62 | | 0.24 | |  | | 10.37 | | 6.74 | |  | 8.26 | | | 6.57 | |  | | |
| **Health condition sample only: Respiratory conditions** | | | | | | | | | | | | | | | | | | | | | | | | | | | | | | | | | | | | | |  | | |  | |  | | |
| No symptoms | 190 | 50.46 | 18.10 | -0.93 | 18.33 | 7.28 | -0.90 | | 0.74 | | 0.25 | | 0.91 | | 24.52 | | 5.27 | | 0.56 | | 0.70 | | 0.26 | | 0.80 | | 0.78 | | 0.21 | | 0.79 | | 5.57 | | 6.70 | | -0.89 | 4.52 | | | 5.49 | | -0.56 | | |
| At least 3 symptoms | 40 | 67.38 | 19.78 |  | 24.89 | 7.99 |  | | 0.52 | | 0.32 | |  | | 21.58 | | 5.99 | |  | | 0.50 | | 0.30 | |  | | 0.62 | | 0.25 | |  | | 10.62 | | 6.16 | |  | 7.58 | | | 5.52 | |  | | |
| **Those accessing social care services** | | | | | | | | | | | | | | | | | | | | | | | | | | | | | | | | | | | | |  |  | | |  | |  | | |
| All needs met | 100 | 58.08 | 17.49 | -1.08 | 21.71 | 7.08 | -0.99 | | 0.59 | | 0.26 | | 0.81 | | 24.57 | | 5.27 | | 1.11 | | 0.54 | | 0.26 | | 0.75 | | 0.75 | | 0.18 | | 1.35 | | 7.17 | | 6.51 | | -1.01 | 5.45 | | | 6.17 | | -0.89 | | |
| Some needs, very few or none of needs met | 119 | 76.98 | 17.35 |  | 28.71 | 7.23 |  | | 0.38 | | 0.29 | |  | | 18.71 | | 5.84 | |  | | 0.35 | | 0.28 | |  | | 0.51 | | 0.24 | |  | | 13.76 | | 6.58 | |  | 10.97 | | | 6.23 | |  | | |

^a^ Summative scores ^b^ Utility score

**Cut-off used:** 0.2-0.49 considered small (in red), 0.5-0.79 moderate (amber), >= 0.8 large

Appendix Table 7 Known-group validity for all measures in the health condition sample

|  | | **Mean scores (95% Confidence Interval)** | | | | | | | | | | | | | |
| --- | --- | --- | --- | --- | --- | --- | --- | --- | --- | --- | --- | --- | --- | --- | --- |
| **Diabetes (n = 234)** [*Hypothesis -Those with higher number of complications have lower health and QoL*] | | | | | | | | | | | | | | | |
| **Number of complications** | **n** | **EQHWB-25** | **EQ-HWB-9** | **SWEMWBS** | | **EQ-5D-5L** | | **ICECAP-A** | **PHQ-8** | | | **GAD-7** | | |  |
| 0 | 169 | 46.75(44.04, 49.45) | 16.79(15.70, 17.87) | 25.51(24.60, 26.43) | | 0.72(0.69, 0.76) | | 0.80(0.76, 0.83) | | 4.76(3.85, 5.68) | | | 3.30(2.48, 4.11) | | |
| 1 | 37 | 55.11(48.44, 61.78) | 19.78(17.02, 22.55) | 24.73(22.79, 26.67) | | 0.63(0.54, 0.71) | | 0.74(0.67, 0.81) | | 6.95(4.81, 9.08) | | | 5.38(3.44, 7.31) | | |
| 2 | 19 | 57.95(48.89, 67.01) | 21.21(17.42, 25.00) | 24.47(21.99, 26.96) | | 0.57(0.46, 0.68) | | 0.75(0.63, 0.86) | | 8.42(5.05, 11.79) | | | 6.21(3.26, 9.16) | | |
| 3 | 6 | 68.33(50.28, 86.38) | 24.67(18.21, 31.12) | 23.83(16.95, 30.71) | | 0.40(0.09, 0.70) | | 0.61(0.39, 0.84) | | 11.83(3.51, 20.16) | | | 7.17(0.79, 13.55) | | |
| 4 | 3 | 79.00(23.01, 105) | 31.33(5.96, 43) | 14.67(0, 21) | | 0.13(-1.47,1) | | 0.48(-0.56,1) | | 13.00(-4, 21) | | | 11.00(-11, 21) | | |
| *p-value (Prob >F)^a^* |  | 0.0001 | 0.0001 | 0.0343 | <0.0001 | | 0.0186 | | 0.0063 | | 0.044 | | |  |  |
| **Arthritis (n = 237 )** [*Hypothesis -Those with higher number of joints affected have lower health and QoL*] | | | | | | | | | | | | |  |  |  |
| Number of joints affected | **Mean scores (95% Confidence Interval)** | | | | | | | | | | | |  |  |  |
| 1 | 45 | 48.51(42.67, 54.35) | 17.13(14.92, 19.35) | 26.18(24.79,27.57) | 0.71(0.65,0.77) | | 0.84(0.79,0.89) | | 4.13(2.36,5.91) | | 2.78(1.34,4.22) | | |  |  |
| 2 | 59 | 53.14(48.61, 57.66) | 19.00(17.17,20.83) | 24.66(23.27,26.06) | 0.62(0.56,0.67) | | 0.79(0.74,0.84) | | 5.76(4.42,7.10) | | 4.31(3.03,5.59) | | |  |  |
| 3 | 49 | 60.14(54.53,65.76) | 21.73(19.48, 23.99) | 23.18(21.41,24.96) | 0.55(0.49,0.61) | | 0.70(0.64,0.77) | | 7.82(5.96,9.67) | | 5.04(3.43,6.65) | | |  |  |
| 4 | 45 | 66.82(61.12,72.52) | 24.58(22.20, 26.96) | 20.80(19.06,22.54) | 0.46(0.37,0.54) | | 0.60(0.53,0.68) | | 9.13(7.21,11.06) | | 5.98(4.29,7.67) | | |  |  |
| 5 | 20 | 72.75(63.58,81.92) | 26.20(22.49,29.91) | 21.25(18.13,24.37) | 0.42(0.29,0.55) | | 0.60(0.48,0.71) | | 10.70(7.55,13.85) | | 8.35(4.92,11.78) | | |  |  |
| 6 | 13 | 67.69(57.33,78.06) | 25.15(20.77, 29.54) | 22.38(19.26,25.51) | 0.47(0.36,0.58) | | 0.66(0.53,0.79) | | 9.31(5.44,13.18) | | 5.85(2.65,9.04) | | |  |  |
| 7 | 6 | 76.33(57.42, 95.24) | 28.17(19.76, 36.57) | 19.50(12.94,26.06) | 0.36(-0.34,0.75) | | 0.54(0.23,0.85) | | 12.00(3.79,20.21) | | 10.67(2.21,19.12) | | |  |  |
| *p-value ^a^* |  | 0.0001 | 0.0001 | 0.0004 | 0.0001 | | 0.0001 | | 0.0001 | | 0.0017 | | |  |  |
| Correlation with arthritis VAS | 237 | 0.51 | 0.50 | -0.32 | -0.57 | | -0.37 | | 0.42 | | 0.32 | | |  |  |
| **Rheumatoid arthritis (n = 46)** [*Hypothesis - those with higher severity index calculated across 5 questions have lower health and QoL*] | | | | | | | | | | | | |  |  |  |
| Correlation with index of severity ^b^ | 46 | 0.60 | 0.59 | -0.27 | -0.41 | | -0.31 | | 0.50 | | 0.40 | | |  |  |
| **Respiratory conditions** | **Standardised effect size** | | | | | | | | | | | |  |  |  |
| No symptoms | 141 | -1.08 | -0.99 | 0.63 | 0.75 | | 0.90 | | -0.97 | | -0.71 | | |  |  |
| At least one symptom | 97 |  |  |  |  | |  | |  | |  | | |  |  |
| ^a^ P-values relate to the joint test of equality ^b^ Summed across pain, active, tenderness and swelling, pain, general health, joint stiffness – this is convergent validity rather than known-group difference. | | | | | | | | | | | | |  |  |  |
|  | | | | | | | | | | | | |  |  |  |

**Appendix Table 8 Test-test reliability at score level**

| **Measure** | **ICC [95% Confidence interval]** | |
| --- | --- | --- |
|  | **Health condition sample** | **General population sample** |
| EQ-HWB-25 sum score | 0.900 [0.862 to 0.927] | 0.931 [0.871 to 0.963) |
| EQ-HWB-9 sum scores | 0.879 [0.847 to 0.905] | 0.876 [0.791 to 0.928] |
| EQ-HWB utility score | 0.866 [0.817 to 0.902] | 0.933 [0.875 to 0.964] |
| SWEMWBS total score | 0.863 [0.817 to 0.898] | 0.893 [0.804 to 0.943] |
| EQ-5D-5L | 0.832 [0.795 to 0.864] | 0.769 [0.659 to 0.855] |

**Appendix Table 9 Test-retest reliability for EQ-HWB items – percentage agreement and Kappa scores**

| EQHWB-25 item | | **Health condition sample** | |  |  | **General population sample** | |  |  |
| --- | --- | --- | --- | --- | --- | --- | --- | --- | --- |
| No | Description | **Agreement** | **Expected agreement** | **Kappa** | **Std error** | **Agreement** | **Expected agreement** | **Kappa** | **Std error** |
| 1 | Seeing | 71.20% | 47.02% | 0.456 | 0.047 | 84.00% | 64.60% | 0.548 | 0.107 |
| 2 | Hearing | 78.80% | 55.21% | 0.527 | 0.045 | 88.00% | 62.00% | 0.684 | 0.107 |
| 3 | Getting around inside and outside* | 73.20% | 40.26% | 0.551 | 0.041 | 88.00% | 78.00% | 0.455 | 0.103 |
| 4 | Day to day activities* | 67.60% | 32.13% | 0.523 | 0.039 | 84.00% | 70.16% | 0.464 | 0.104 |
| 5 | Washing, eating, dressing, caring | 79.60% | 55.28% | 0.544 | 0.043 | 94.00% | 88.48% | 0.479 | 0.103 |
| 6 | Problems with sleep | 56.40% | 20.52% | 0.451 | 0.032 | 58.00% | 31.84% | 0.384 | 0.091 |
| 7 | Exhausted* | 55.20% | 21.02% | 0.433 | 0.032 | 54.00% | 33.96% | 0.304 | 0.091 |
| 8 | Lonely * | 70.80% | 33.29% | 0.562 | 0.037 | 66.00% | 52.24% | 0.288 | 0.094 |
| 9 | Unsupported by people | 65.20% | 35.72% | 0.459 | 0.038 | 70.00% | 51.88% | 0.377 | 0.091 |
| 10 | Trouble remembering | 66.00% | 30.34% | 0.512 | 0.038 | 74.00% | 57.24% | 0.392 | 0.119 |
| 11 | Trouble concentrating* | 61.60% | 30.42% | 0.448 | 0.037 | 78.00% | 53.80% | 0.524 | 0.107 |
| 12 | Anxious* | 60.40% | 24.54% | 0.475 | 0.034 | 78.00% | 43.04% | 0.614 | 0.101 |
| 13 | Unsafe | 74.40% | 52.25% | 0.464 | 0.041 | 84.00% | 74.88% | 0.363 | 0.108 |
| 14 | Frustrated | 56.80% | 24.82% | 0.425 | 0.035 | 66.00% | 36.68% | 0.463 | 0.096 |
| 15 | Sad or depressed* | 58.80% | 25.94% | 0.444 | 0.035 | 82.00% | 47.48% | 0.657 | 0.091 |
| 16 | Nothing to look forward to | 61.60% | 28.22% | 0.465 | 0.035 | 82.00% | 54.16% | 0.607 | 0.089 |
| 17 | No control over day to day* | 62.40% | 31.27% | 0.453 | 0.037 | 66.00% | 50.24% | 0.317 | 0.087 |
| 18 | Unable to cope with my day | 68.00% | 38.30% | 0.481 | 0.040 | 86.00% | 65.72% | 0.592 | 0.102 |
| 19 | Accepted by others | 54.00% | 24.55% | 0.390 | 0.035 | 58.00% | 30.44% | 0.396 | 0.081 |
| 20 | Good about myself | 56.80% | 21.30% | 0.451 | 0.032 | 56.00% | 24.16% | 0.420 | 0.076 |
| 21 | Do the things I wanted to do | 57.60% | 22.79% | 0.451 | 0.034 | 60.00% | 30.52% | 0.424 | 0.085 |
| 22 | Physical pain (frequency) | 54.40% | 21.12% | 0.422 | 0.032 | 54.00% | 31.08% | 0.333 | 0.088 |
| 23 | Physical pain (severity)* | 66.00% | 28.81% | 0.522 | 0.038 | 68.00% | 37.56% | 0.488 | 0.102 |
| 24 | Physical discomfort (frequency) | 50.00% | 24.77% | 0.335 | 0.035 | 72.00% | 49.36% | 0.447 | 0.109 |
| 25 | Physical discomfort (severity) | 56.40% | 29.10% | 0.385 | 0.039 | 82.00% | 46.80% | 0.662 | 0.108 |

Note: p-value is <0.0001 for all items

**Appendix Figure 1 EQ-HWB-25 total scores at baseline by health condition and general population samples**

**Appendix Figure 2 EQ-HWB-9 sum scores at baseline by health condition and general population samples**

**Appendix Figure 3 SWEMWBS total scores at baseline by health condition and general population samples**

Appendix Figure 4 EQ-5D-5L scores at baseline by health condition and general population samples

**Appendix Figure 5 ICECAP-A tariffs at baseline by health condition and general population samples**

**Appendix Figure 6 PHQ-8 at baseline by health condition and general population samples**

**Appendix Figure 7 GAD-7 at baseline by health condition and general population samples**
